# Induction of the zinc finger transcription factor GATA2 promotes kidney inflammation-related gene expression

**DOI:** 10.1016/j.jbc.2025.110372

**Published:** 2025-06-16

**Authors:** Jun Takai, Hinata Ueki, Satoshi Uemura

**Affiliations:** Faculty of Medicine, Division of Medical Biochemistry, Tohoku Medical and Pharmaceutical University, Sendai, Miyagi, Japan

**Keywords:** ATAC-seq, chromatin accessibility, CUT&Tag, *Csf1*, *Cxcl10*, GATA2, GATA transcription factor, inflammation, kidney, *Vcam1*

## Abstract

Kidney diseases pose a medical challenge worldwide. Excessive kidney inflammation plays a central role in disease progression. While the transcription factor GATA binding protein 2 (GATA2) is known to govern the hematopoietic system, emerging evidence suggests that it also promotes kidney inflammation. To date, the precise molecular mechanisms underlying GATA2-mediated kidney inflammation remain unclear. Here, we examined the transcriptional landscape, genome-wide GATA2 occupancy, and chromatin accessibility upon GATA2 induction in kidney cells. We generated an inducible GATA2 expression system using a renal tubular cell line and then performed RNA-seq, Assay for Transposase-Accessible Chromatin (ATAC)-seq, and Cleavage Under Targets and Tagmentation (CUT&Tag). We also conducted ATAC-seq using GATA2-expressing cell fractions sorted from mouse kidney tissues. These comprehensive analyses demonstrated that GATA2 directly upregulates genes associated with kidney inflammation. In particular, GATA2 bound to crucial kidney inflammation-associated gene loci and increased chromatin accessibility at these regions, including colony-stimulating factor 1 (*Csf1*), C-X-C motif chemokine ligand 10 (*Cxcl10*), and vascular cell adhesion molecule-1 (*Vcam1*). Motif analysis revealed that the binding sequences of the activator protein-1 (AP-1), an inflammation-induced transcription factor, are frequently located adjacent to genomic regions where GATA2 increases chromatin accessibility. Furthermore, the upregulation of *Csf1*, *Cxcl10*, and *Vcam1* following GATA2 induction was attenuated by the AP-1-specific inhibitor T-5224. Overall, this study is the first to determine genome-wide GATA2 occupancy and its impact on chromatin accessibility in kidney cells. These findings provide molecular insights into the role of GATA2 in kidney inflammation.

The kidney is an essential organ responsible for eliminating waste products and maintaining water homeostasis; it is also susceptible to damage from excessive inflammation. Approximately 850 million people worldwide suffer from kidney diseases, including acute kidney injury (AKI) and chronic kidney disease (CKD), both of which have high morbidity and mortality rates and are increasingly recognized as major health concerns (1, 2). Because kidney inflammation plays a key role in both AKI and CKD, elucidating the underlying molecular mechanisms is crucial to better understand their pathophysiology (3, 4). In 2017, we were the first to report that GATA binding protein 2 (GATA2) promotes kidney inflammation (5).

GATA2 is a member of the GATA family of transcription factors, characterized by two C4 zinc fingers that function as its DNA-binding domain and recognize the consensus motif WGATAR (6, 7, 8, 9, 10, 11, 12). Early studies using *Gata2* knock out (KO) mice and *Gata2*-GFP knock-in (KI) mice have demonstrated that GATA2 is expressed in hematopoietic stem and progenitor cells, where it governs hematopoietic development (13, 14, 15, 16). GATA2 is now widely recognized to be expressed and to function in mature hematopoietic cells, including mast cells and basophils, as well as in non-hematopoietic tissues such as endothelial cells, the pituitary gland, and the inner ear, all of which are committed to specific lineages (14, 17, 18, 19, 20, 21, 22, 23, 24, 25, 26). As for mature kidney tissues, we and others have previously reported that GATA2 is expressed specifically in the renal collecting duct, where it plays a crucial role in maintaining water homeostasis by regulating the aquaporin 2 (*Aqp2*) and arginine vasopressin receptor 2 (*Avpr2*) genes (27, 28). We have also shown that mice with renal tubular cell-specific *Gata2* deletion exhibit less severe kidney damage and reduced levels of inflammatory cytokines during renal ischemia–reperfusion injury (5). These data indicate that GATA2 exacerbates kidney injury by promoting kidney inflammation. Several laboratories have subsequently reported that GATA2 participates in kidney inflammation and kidney injury (29, 30, 31), highlighting its potential as a key regulator. To date, the precise molecular mechanisms underlying GATA2-mediated kidney inflammation remain obscure.

Chromatin accessibility changes dynamically across various cell types and pathophysiological conditions, and its increase promotes transcriptional activation (32, 33). Transcription factors (TFs) are recognized as key regulators of chromatin accessibility. Comprehensive analyses of chromatin accessibility and TF occupancy thus provide molecular insights into the transcriptional activation mediated by a TF of interest. Assay for Transposase-Accessible Chromatin (ATAC-seq) is a genome-wide method for analyzing chromatin accessibility (34, 35). Cleavage Under Targets and Tagmentation (CUT&Tag) is a recently developed technique for genome-wide profiling of histone modifications and TF occupancy at high resolution (36), offering an alternative to Chromatin Immunoprecipitation Sequencing (ChIP-seq). Despite the growing number of studies using these genome-wide methods, no analyses have been conducted on GATA2 occupancy and chromatin accessibility in kidney cells.

To investigate this issue, we performed RNA-seq, ATAC-seq, and GATA2 CUT&Tag using newly established kidney cell lines with inducible GATA2 expression. Induction of GATA2 increased both mRNA expression and chromatin accessibility in genes associated with kidney inflammation. We further confirmed that these results are consistent with ATAC-seq data from mouse kidney tissues. Collectively, this study provides the first analysis of genome-wide GATA2 occupancy and its impact on chromatin accessibility in kidney cells, offering molecular insights into the role of GATA2 in kidney inflammation.

## Results

### Generation of a stable cell line with doxycycline-inducible GATA2 expression vector derived from a mouse renal tubular cell line

Inducible gene systems offer a straightforward approach to directly assess whether GATA2 comprehensively regulates transcription and chromatin accessibility in genes associated with kidney inflammation. We first generated a stable cell line with a doxycycline (dox)-inducible GATA2 expression vector to explore how GATA2 accelerates kidney inflammation. We found that mIMCD-3 (an mouse inner medullary collecting duct cell line) exhibited only marginal expression of GATA2 and other GATA TFs based on published RNA-seq data (37) (Fig. S1*A*). NIH3T3 cells (a murine embryo fibroblast cell line) exhibited moderate *Gata2* expression (38), while P815 mouse mastocytoma cell lines exhibited high *Gata2* expression (Fig. S1, *B* and *C*). We found that GATA2 protein levels were consistent with these results by our flow cytometry analysis (Fig. S1, *D* and *E*). Based on these findings, we utilized the mIMCD-3 cell line as a dox-inducible GATA2 expression system.

**Figure 1:**
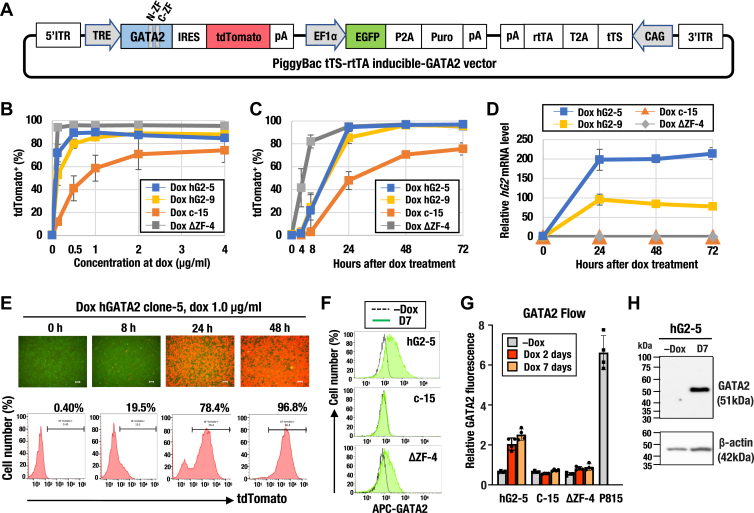
**Generation of a stable cell line with doxycycline (dox)-inducible GATA2 derived from a mouse renal tubular cell line.***A*, construct of the PiggyBac tTS-rtTA inducible-GATA2 vector used in this study. *B*, percentage of tdTomato-positive cells 24 h after dox treatment at various dox concentrations analyzed by flow cytometry (n = 3 per construct). *C*, percentage of tdTomato-positive cells after dox treatment analyzed by flow cytometry (n = 4 per construct). *D*, relative exogenous *hGATA2* (*hG2*) mRNA levels quantified by RT-qPCR using a *hG2*-specific primer targeting the C-ZF domain (n = 3 per group). No increase in *hG2* expression was observed in the C-15 or ΔZF-4 cells after dox treatment. *E*, representative fluorescence images (*upper panel*) and histograms of tdTomato fluorescence (*lower panel*) in the dox hG2-5 cell line after dox treatment. Scale bar: 100 μm. *F*, representative histograms of GATA2 fluorescence analyzed by flow cytometry without dox (−Dox) or 7 days after dox treatment (D7). *G*, relative GATA2 fluorescence levels quantified by flow cytometry (n = 4 per group) at −Dox, 2 days, and 7 days after dox treatment. Values were normalized to the isotype IgG control. P815 was used as a GATA2-positive control. *H*, immunoblot analysis of GATA2 and β-actin in the dox hG2-5 cell line at −Dox or D7. Data from independent replicates are plotted in the bar graph. All data are presented as means ± SD in the graph.

We next designed a novel all-in-one dox-inducible expression vector (PiggyBac tTS-rtTA inducible-GOI vector) by combining a reverse tetracycline-controlled transactivator (rtTA) with a tetracycline transcriptional silencer (tTS) (Fig. S2*A*). This system provided a high signal-to-noise ratio with minimal leakage in the absence of dox (39) (Fig. S2*B*). Using this vector, we established stable cell lines expressing human full-length GATA2 cDNA (hGATA2 or hG2), a GATA2 cDNA lacking both zinc finger domains (ΔZF), or the empty vector (control) (Figs. 1*A*; S2*A*). Human and mouse GATA2 proteins share 97.9% sequence similarity, while their cDNA sequences differ slightly, with 89.3% similarity. Therefore, we used hGATA2 to detect exogenous *Gata2* using a primer specific to *hG2* (see also *Experimental procedures* for details). After selecting each cell line (Fig. S2*C*), we employed hGATA2 clone-5 (dox hG2-5), hGATA2 clone-9 (dox hG2-9), control clone-15 (dox c-15), and ΔZF clone-4 (dox ΔZF-4) for subsequent analyses. We observed that tdTomato-positive cells reached saturation when treated with 1.0 μg/ml dox for 24 h (Fig. 1*B*). All subsequent experiments were thus performed at this concentration. Dox treatment increased the percentage of tdTomato-positive cells in a time-dependent manner, and in dox hG2-5, almost all cells (approximately 97%) became tdTomato-positive within 48 h after dox treatment (Fig. 1, *C* and *E*). Exogenous *Gata2* mRNA was induced by dox treatment in both hG2-5 and hG2-9 cells, as confirmed by RT-qPCR using a *hG2*-specific primer targeting the C-terminal zinc finger (C-ZF) domain (Fig. 1*D*). Flow cytometry and Western blotting analyses also confirmed GATA2 protein expression at 2 and 7 days following dox treatment (Figs. 1, *F*–*H*; S2, *D* and *E*). In conclusion, we successfully generated stable cell lines with dox-inducible GATA2 expression.

### Induction of GATA2 upregulates Il6 expression and increases chromatin accessibility at its promoter

**Figure 2:**
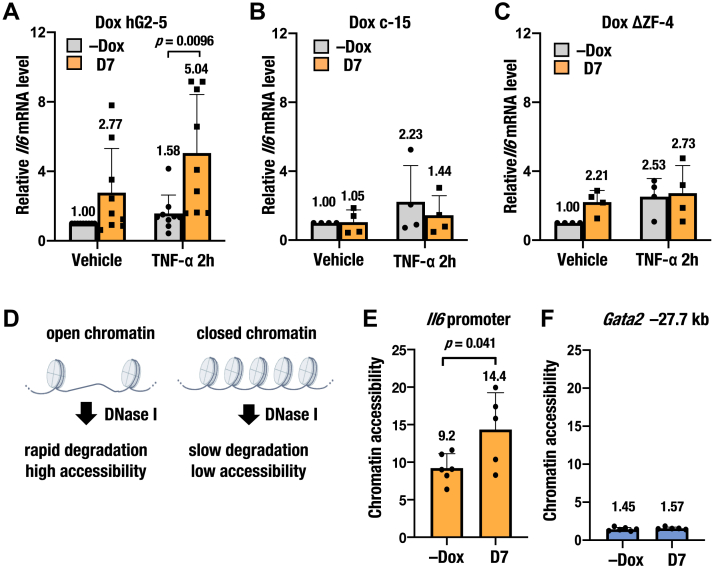
**Upregulated *Il6* expression and increased chromatin accessibility at *Il6* promoter *via* GATA2 induction.***A*–*C*, relative *Il6* mRNA levels quantified by RT-qPCR without dox (−Dox) and 7 days after dox treatment (D7). The mRNA levels were normalized to *Polr2a*, with vehicle-treated −Dox group defined as 1.0 (n = 9 for dox hG2-5, n = 4 for dox control-15 and dox ΔZF-4). TNF-α was added to stimulate inflammatory response. *D*, experimental principle of DNase I chromatin accessibility analysis. Open chromatin with *high* chromatin accessibility is more susceptible to DNase I digestion, resulting in *lower* DNA content. Chromatin accessibility is thus quantified by qPCR. See Experimental procedures for details. *E* and *F*, chromatin accessibility at the *Il6* promoter and the *Gata2* −27.7k genomic region in the dox hG2-5 cell (n = 6 for −Dox group, n = 5 for D7 group). The *Gata2* −27.7k region was analyzed as a control genomic region with *low* chromatin accessibility. Data from independent replicates are plotted in the bar graph. All data are presented as means ± SD in the graph. *p*-values were calculated using unpaired t-tests.

We and others previously showed that GATA2 knockdown reduces interleukin-6 (*Il6*) expression and decreases chromatin accessibility in mast cells (40, 41). We therefore examined whether inducible GATA2 expression could upregulate *Il6* expression and increase chromatin accessibility using our system. Dox-inducible GATA2 expression significantly increased *Il6* mRNA levels at both 2 and 7 days following dox treatment upon tumor necrosis factor-α (TNF-α) stimulation, with a more increase observed at 7 days (Figs. S3*A*; 2*A*). Time-course analysis revealed a peak in *Il6* expression at 2 h upon TNF-α stimulation, which returned to baseline by 24 h (Fig. S3*B*). Accordingly, we selected a 7-day dox treatment and a 2 h TNF-α stimulation for subsequent experiments. This *Il6* upregulation was not observed in either dox c-15 or ΔZF-4 cells (Fig. 2, *B* and *C*). DNase I chromatin accessibility analysis revealed that inducible GATA2 expression significantly increased chromatin accessibility at the *Il6* promoter but not at the *Gata2* −27.7 kb control region (Fig. 2, *D*–*F*). These results indicate that the newly generated cell line is a valuable system for analyzing GATA2-mediated inflammatory responses.

### RNA-seq analysis reveals that GATA2 induction upregulates expression of genes involved in kidney inflammation

**Figure 3:**
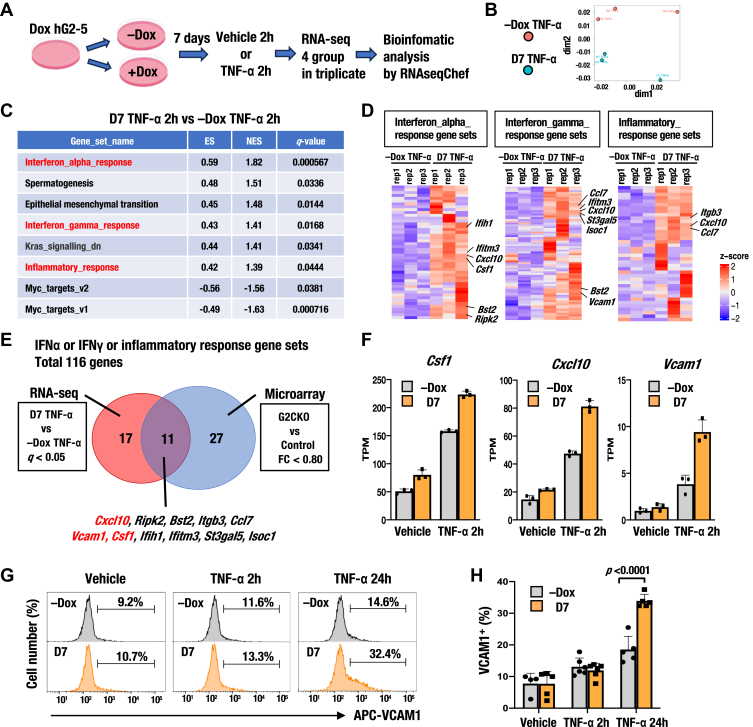
**Upregulation of inflammation-related genes upon inducible GATA2 expression identified by RNA sequencing.***A*, experimental workflow of RNA-seq. *B*, clustering of TNF-α-stimulated groups by multidimensional scaling. *C*, gene Set Enrichment Analysis (GSEA) between + dox 7 days (D7) TNF-α 2h and −Dox TNF-α 2h. Gene sets with *q*-values (adjusted *p*-values for multiple testing) < 0.05 are listed. Gene sets associated with inflammation are depicted in *red*. *D*, heatmaps displaying gene sets for Interferon_alpha_response, Interferon_gamma_response, and Inflammatory_response in D7 TNF-α 2h and −Dox TNF-α 2h. *E*, Venn diagrams showing GATA2 upregulated genes from RNA-seq data (*red*; this study) and downregulated genes from a microarray of G2CKO mice (*blue*; GSE52448). Eleven overlapping genes are extracted and listed. *F*, mRNA expression levels of the *Csf1*, *Cxcl10*, and *Vcam1* genes, quantified by RNA-seq (n = 3 per group). *G*, representative histograms of VCAM1 fluorescence analyzed by flow cytometry. *H*, percentage of VCAM1-positive cells in hG2-5 cells at −Dox and D7 after TNF-α stimulation, analyzed by flow cytometry (n = 4–6 per group). Data from independent replicates are plotted in the bar graph. All data are presented as means ± SD in the graph. Control, wild-type mice; ES, enrichment score; G2CKO, renal tubule-specific GATA2-deficient mice; NES, normalized enrichment score.

To dissect the transcriptional landscape of inflammation-associated genes upregulated by GATA2, we performed RNA-seq using dox hG2-5 cells divided into four groups: −Dox vehicle, +dox 7 days (D7) vehicle, −Dox TNF-α, and +dox 7 days (D7) TNF-α, each in triplicate (Figs. 3, *A* and *B*; S3*C*). Dox treatment increased *Gata2* mRNA levels regardless of TNF-α stimulation (as expected) but did not affect mRNA levels of other GATA TFs (Fig. S3*D*). *Il6* expression seemed to align with the trend observed in manual RT-qPCR analysis, although some replicates were below the detection limit due to low expression levels (Fig. S3*E*). Gene set enrichment analysis (GSEA) revealed that inducible GATA2 expression led to the upregulation of gene sets involved in interferon-alpha, interferon-gamma, and inflammatory response upon TNF-α stimulation (Figs. 3*C*; S3*F*). These upregulated gene sets were visualized using heat maps (Figs. 3*D*; S3*G*).

To further narrow the set of GATA2-dependent genes, we combined our current RNA-seq results with previously reported microarray data from renal tubule-specific GATA2-deficient mice (5). We identified 11 genes from the interferon-alpha (IFNα), interferon-gamma (IFNγ), and inflammatory response gene sets (total: 116 genes) with adjusted *p*-values < 0.05 in RNA-seq and downregulated by more than 20% due to GATA2 loss in the microarray data (Fig. 3*E*). Colony-stimulating factor 1 (*Csf1*) and C-X-C motif chemokine ligand 10 (*Cxcl10*) are expressed in renal epithelial cells and play crucial roles in producing and infiltrating inflammatory cells, thereby contributing to kidney disease development (42, 43). Vascular cell adhesion molecule-1 (*Vcam1*) is reportedly upregulated during kidney injury, promotes renal failure, and shows potential as a biomarker for kidney disease (44, 45, 46). We confirmed that these three genes were upregulated by inducible GATA2 expression upon TNF-α stimulation, with consistent effects observed across replicates (Fig. 3*F*). In addition, GATA2 induction significantly increased the percentage of VCAM1-positive cells 24 h after TNF-α stimulation (Fig. 3, *G* and *H*). These findings prompted us to focus on the *Csf1*, *Cxcl10,* and *Vcam1* genes in subsequent analysis.

### Optimization of GATA2 CUT&Tag

We next investigated the genome-wide landscape of GATA2 occupancy and chromatin accessibility following inducible GATA2 expression. Unlike ATAC-seq, CUT&Tag utilizes validated antibodies, necessitating target-specific optimization. To date, only a few studies have reported CUT&Tag using a GATA2 antibody (47, 48, 49). One study found that CUT&Tag with a commercially available GATA2 antibody failed to enrich the GATA motif. We thus optimized our experimental conditions for CUT&Tag using three commercially available GATA2 antibodies in P815 cells that highly expresses GATA2 (Fig. S1, *C*–*E*).

**Figure 4:**
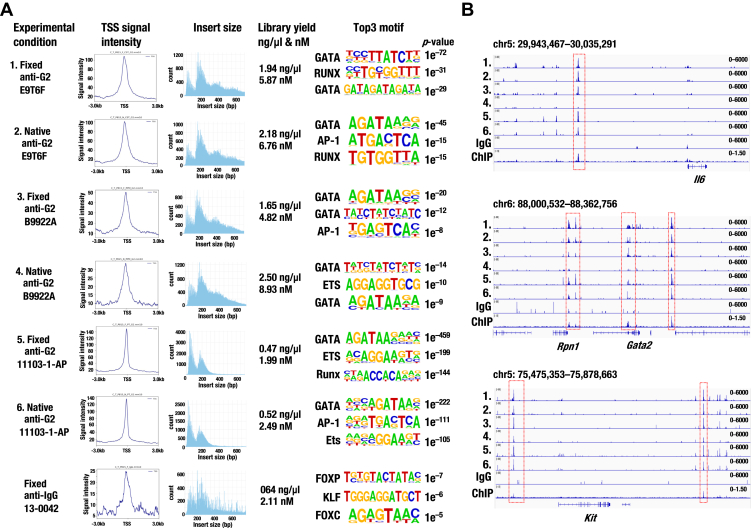
**Optimization of GATA2 CUT&Tag in P815 cells.***A*, quality control (QC) results for six CUT&Tag experimental conditions using combinations of three commercially available GATA2 antibodies (E9T6F, B9922A, and 11103-1-AP) and two cell fixation states (fixed or native). CUT&Tag with an IgG antibody was used as a negative control. Signal intensity at the transcription start site (TSS), insert size distribution, library yield per 10^5^ cells, and *top* three enriched motifs identified by HOMER are shown. *p*-values calculated *via* the hypergeometric test are depicted. *B*, representative GATA2 binding peaks at *Il6*, *Gata2*, and *Kit* loci visualized using Integrative Genomics Viewer (IGV). Numbers 1–6 on the *left* correspond to GATA2 CUT&Tag peaks under each condition in Figure 4*A*. IgG indicates peaks generated from the CUT&Tag library with IgG. ChIP represents previously published GATA2 ChIP-seq data (PRJDB12807). Reads Per Kilobase of exon per Million mapped reads (RPKM) values on the Y-axis are shown on the *right* of each track. Overlapping peaks between GATA2 CUT&Tag and ChIP-seq are indicated by *red dotted lines*.

All CUT&Tag libraries, except for control IgG, showed a nucleosomal ladder pattern in insert size distribution and enrichment of the GATA motif (Fig. 4*A*). Cell fixation resulted in slightly lower library yields than native conditions but modestly increased transcription start site (TSS) intensity across all antibodies (Fig. 4*A*). The ChIP-seq-validated B9922A antibody unexpectedly exhibited a low TSS intensity under native cell conditions (Fig. 4*A*). In contrast, the E9T6F and 11103-1-AP antibodies exhibited high TSS signals regardless of whether the cells were in native or fixed conditions (Fig. 4*A*). The Integrative Genomics Viewer (IGV) track data at the *Il6*, *Gata2*, and *Kit* loci were consistent with these CUT&Tag quality control (QC) results (Fig. 4*B*). Overall, the CUT &Tag data obtained using E9T6F and 11103-1-AP antibodies exhibited a higher signal-to-noise ratio than previously published GATA2 ChIP-seq data at these loci (Fig. 4*B*). Among the two antibodies, E9T6F yielded a higher library output than 11103-1-AP, and its quality is likely to remain stable due to its monoclonal nature. Given these advantages, we selected E9T6F for CUT&Tag in this study.

### GATA2 CUT&Tag and ATAC-seq analyses reveal that GATA2 induction increases GATA2 binding and chromatin accessibility at Csf1, Cxcl10, and Vcam1 loci

**Figure 5:**
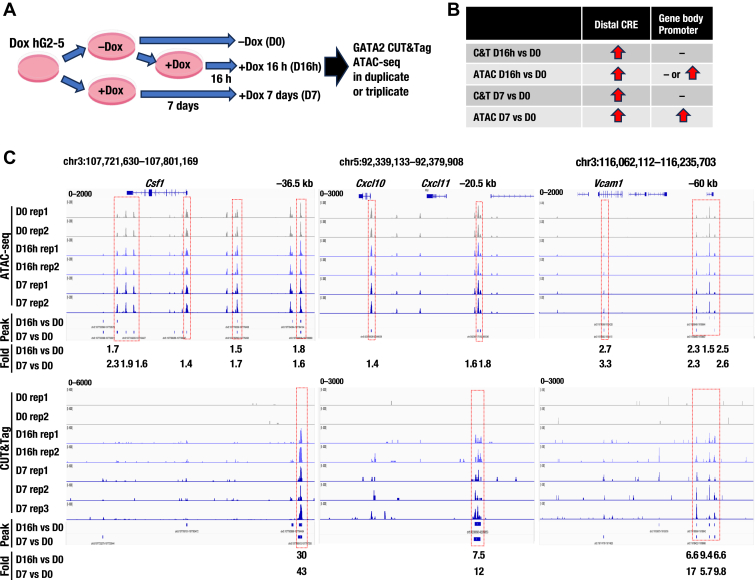
**GATA2 binding and increased chromatin accessibility at the *Csf1*, *Cxcl10*, and *Vcam1* loci upon GATA2 induction.***A*, experimental workflow for GATA2 CUT&Tag and ATAC-seq. *B*, summary of GATA2 CUT&Tag and ATAC-seq results at the *Csf1*, *Cxcl10,* and *Vcam1* loci. *C*, representative ATAC-seq peaks and GATA2 binding peaks at the *Csf1*, *Cxcl10,* and *Vcam1* loci visualized using IGV. Peaks indicate significantly increased regions at D16h or D7 compared to D0 (*p* < 1e−5). *p*-values were calculated using a Poisson distribution. Fold represents fold enrichment of D7 or D16h relative to D0. Significantly increased chromatin accessibility regions from ATAC-seq (*upper panel*) and GATA2-binding regions from CUT&Tag (*lower panel*) are indicated by *red dotted lines*. RPKM values on the Y-axis are shown in the *upper left* of each track.

Based on our chromatin accessibility assay results (Fig. 2, *E* and *F*), we hypothesized that GATA2 acts on chromatin prior to inflammation. To further investigate this hypothesis, we performed ATAC-seq and GATA2 CUT&Tag using the dox hG2-5 cell line, which was divided into three groups according to the different stages of GATA2 induction: a D0h control group, a D16h early-stage group, and a D7 late-stage group (Fig. 5*A*). QC analysis confirmed the reliability of these experiments (Fig. S4, *A* and *B*).

We found that GATA2 initially binds to distal cis-regulatory elements (CREs) of the *Csf1*, *Cxcl10*, and *Vcam1* loci and enhances pre-existing chromatin accessibility around these genomic regions. As a result, chromatin accessibility to these promoters and gene bodies increases at D16h and D7 (Fig. 5*B*). For example, at the *Csf1* locus, GATA2 bound to the *Csf1* −36.5 kb region at D16h and D7, indicating persistent GATA2 binding to this region (Fig. 5*C*). GATA2 binding significantly increased chromatin accessibility at the *Csf1* −36.5 kb region by 1.6–1.8-fold compared to that at D0 (Fig. 5*C*). Chromatin accessibility was significantly increased at the gene body and promoter of the *Csf1* locus, particularly in D7 (Fig. 5*C*). Similar results were observed for *Cxcl10* and *Vcam1* loci (Fig. 5*C*). Among the 11 gene loci identified in Figuer 3, four loci (*Csf1, Itgb3, Ripk2,* and *Bst2*) exhibited shared GATA2 binding peaks in both P815 and hG2-5 cells (Fig. S4*C*). Taken together, the CUT&Tag and ATAC-seq analyses indicate that GATA2 directly binds to kidney inflammation-associated gene loci and that GATA2 induction enhances pre-existing chromatin accessibility at these loci in hG2-5 cells.

### ATAC-seq analysis reveals that GATA2-expressing kidney cells exhibit an accessible chromatin at *Csf1*, *Cxcl10*, and *Vcam1* loci in mouse kidney tissues

**Figure 6:**
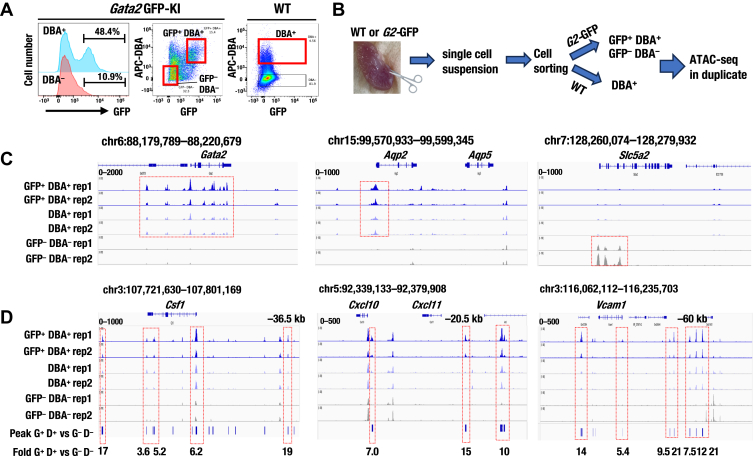
**Increased chromatin accessibility at the *Csf1*, *Cxcl10*, and *Vcam1* loci in GATA2-expressing mouse kidney tissues.***A*, GFP histogram and dot plots from the kidney of *Gata2*-GFP knock-in mice (*Gata2*-GFP KI) and wild-type (WT). The fraction used for ATAC-seq is circled with a *red line*. DBA (Dolichos biflorus agglutinin) lectin is a marker for the renal collecting duct. The dot plots shown in *panel A* and the corresponding detailed gating strategies are also depicted in Figure S9*B*. *B*, experimental workflow of ATAC-seq. *C*, representative ATAC-seq peaks at *Gata2*, *Aqp2,* and *Slc5a2* loci visualized using IGV. Specific peaks at the *Gata2* and *Aqp2* loci in the GATA2-enriched fractions (GFP^+^ DBA^+^ or DBA^+^) and at the *Slc5a2* locus in the GATA2-depleted fraction (GFP^−^ DBA^−^) are indicated by *red dotted lines*. *D*, representative ATAC-seq peaks at the *Csf1*, *Cxcl10,* and *Vcam1* loci visualized using IGV. Peaks indicate significantly increased regions in GFP^+^ DBA^+^ compared to GFP^−^ DBA^−^ (*p* < 1e−5). *p*-values were calculated using a Poisson distribution. Fold represents fold enrichment of GFP^+^ DBA^+^ relative to GFP^−^ DBA^−^. Significantly increased chromatin accessibility regions from ATAC-seq are indicated by *red dotted lines*.

We next performed ATAC-seq analysis using mouse kidney tissues to investigate whether the chromatin accessibility peaks identified in the hG2-5 cell line can also be observed under physiological conditions. Previously, we reported that renal GATA2-expressing cells could be enriched with Dolichos biflorus agglutinin (DBA) (28). As expected, flow cytometry analysis revealed that approximately 50% of DBA-positive kidney cells exhibited GFP fluorescence in *Gata2*-GFP knock-in (KI) mice (Fig. 6*A*). Based on this, we performed ATAC-seq analysis of mouse kidney tissues, categorizing them into three fractions: a GFP^+^ DBA^+^ (GATA2-enriched) fraction, a GFP^−^DBA^−^ (GATA2-depleted) fraction from *Gata2*-GFP KI mice, and a DBA^+^ (GATA2-enriched) fraction from wild-type (WT) mice (Fig. 6, *A* and *B*). QC analysis confirmed the reliability of these ATAC-seq results (Fig. S5). As anticipated, specific chromatin accessibility peaks around the *Gata2* locus were observed in the GATA2-enriched kidney fractions (Fig. 6*C*). Aquaporin 2 *(Aqp2)*, which is expressed in principal cells (PCs) in the renal collecting ducts, showed specific peaks exclusively in the GATA2-enriched kidney fraction (Fig. 6*C*). In contrast, solute carrier family 5 member 2 (*Slc5a2)*, which encodes SGLT2 and is expressed in the renal proximal tubule, showed specific peaks only in the GATA2-depleted kidney fraction (Fig. 6*C*). These results indicate that ATAC-seq analysis of the GATA2-enriched fraction represents chromatin accessibility in renal PCs, whereas ATAC-seq analysis of the GATA2-depleted fraction represents chromatin accessibility in renal proximal tubular cells.

In line with our hG2-5 cell line results, we observed similar chromatin accessibility peaks in the GATA2-enriched kidney fraction at the *Csf1* −36.5 kb, *Cxcl10* −20.5 kb, and *Vcam1* −60 kb regions (Fig. 6*D*). Chromatin accessibility at these loci was significantly higher in the GATA2-enriched fraction compared to the GATA2-depleted fraction, showing a 19-fold increase for *Csf1*, 15-fold for *Cxcl10*, and 7.5- to 21-fold for *Vcam1*. Moreover, accessibility at the promoter regions of these genes was also significantly increased, with a 6.2-fold increase for *Csf1*, 7.0-fold for *Cxcl10*, and 5.4-fold for *Vcam1* (Fig. 6*D*). These data indicate that increased chromatin accessibility at the *Csf1*, *Cxcl10*, and *Vcam1* loci is associated with GATA2 expression in kidney tissues, thereby supporting the physiological relevance of our findings in the hG2-5 cell line.

### AP-1 binding sequences are enriched adjacent to GATA2-binding genomic regions

**Figure 7:**
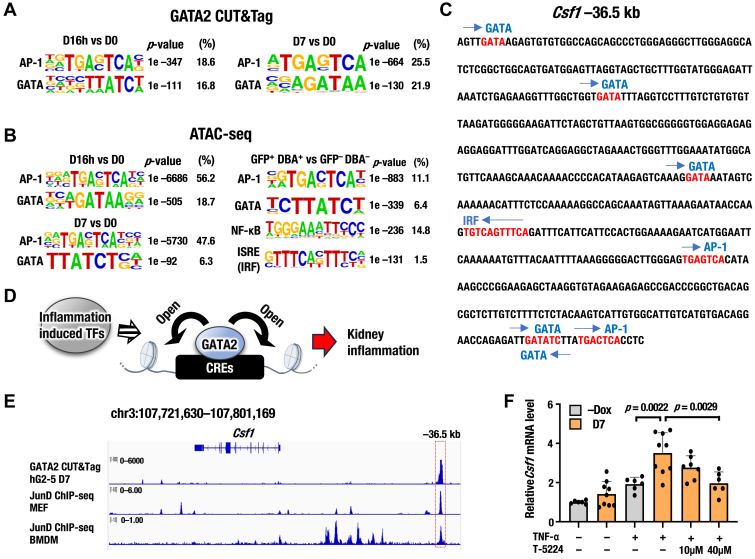
**Enrichment of GATA and inflammation-induced transcription factor binding motifs in GATA2-expressing kidney cells.***A*, enrichment of AP-1 and GATA binding motifs specific to D16h (*left*) or D7 (*right*) compared to D0 in GATA2 CUT&Tag. *B*, *Left panel*: Enrichment of AP-1 and GATA binding motifs specific to D16h (*top*) or D7 (*bottom*) compared to D0 in ATAC-seq using the dox hG2-5 cell line. Right panel: Enrichment of inflammation-induced transcription factors (TFs) and GATA binding motifs specific to GFP^+^ DBA^+^ (GATA2 enriched fraction) compared to GFP^−^ DBA^−^ (GATA2 depleted fraction) in ATAC-seq using mouse kidney tissues. *p*-values were calculated using a hypergeometric test, and the percentages of peaks containing motifs are shown in (*A* and *B*). *C*, nucleotide sequences at *Csf1* −36.5 kb genomic region around the GATA2-binding peak identified by CUT&Tag. Binding motifs for inflammation-induced TFs and GATA are indicated. *D*, proposed model for the molecular mechanisms by which GATA2 accelerates kidney inflammation. GATA2 binds to the cis-regulatory elements (CREs) of inflammation-related genes and opens the chromatin structure that contains binding sites for inflammation-induced TFs. This accessible chromatin environment enhances transcription efficiently by inflammation-induced TFs. *E*, representative GATA2 and JunD binding peaks at the *Csf1* locus. GATA2 CUT&Tag data from hG2-5 cells at D7 and previously reported JunD ChIP-seq data (SRX13793008 and SRX2901296) are shown. *F*, relative *Csf1* mRNA levels at −Dox and D7 in hG2-5 cells, quantified by RT-qPCR (n = 6 for −Dox group and n = 6–9 for D7 group). Cells were treated with TNF-α (20 ng/ml, 2 h) and the AP-1 inhibitor T-5224 (10 or 40 μM, 1 h) prior to analysis. *p*-values in (*F*) were calculated using one-way ANOVA followed by Tukey’s *post hoc* test. Data from independent replicates are plotted, and values are presented as means ± SD in the bar graph. BMDM, bone marrow-derived macrophage; MEF, mouse embryonic fibroblast.

To gain deeper insights into the molecular mechanisms by which GATA2 enhances inflammation-related gene transcription, we performed motif analysis using HOMER. We first extracted the top 20 TF-binding motifs specific to GATA2-expressing cells based on all CUT&Tag and ATAC-seq data generated in this study (Fig. S6). Among these, the GATA motif was enriched as expected (Fig. 7, *A* and *B*). Interestingly, binding motifs for inflammation-induced TFs, including activator protein 1 (AP-1), nuclear factor kappa-light-chain-enhancer of activated B cells (NF-κB), and interferon regulatory factors (IRFs), were also enriched (Fig. 7, *A* and *B*). These results suggest that AP-1, NF-κB, and IRF binding sequences are located in the vicinity of the genomic regions where GATA2 increases chromatin accessibility. Indeed, these TF-binding sequences were identified in proximity to the GATA2-binding site in the *Csf1* −36.5 kb, *Cxcl10* −20.5 kb, and *Vcam1* −60 kb genomic regions (Figs. 7*C*; S7). These findings led us to propose a model in which GATA2 and inflammation-induced TFs cooperatively upregulate kidney inflammation-associated genes (Fig. 7*D*).

To estimate significant TF pathways, we examined the transcript levels of AP-1 (Jun and Fos family), NF-κB, and IRF TFs in hG2-5 cells. We found that *Junb, Jund, Fosl1, Nfkb1, Nfkb2*, and *Rela* were highly expressed (TPM ∼100 or higher) in hG2-5 cells (Fig. S8, *A*–*D*). Among these TFs, *Jund,* a component of AP-1, was the most abundant at the transcript level. JunD protein was specifically expressed in renal collecting ducts and the distal tubule, according to the HUMAN PROTEIN ATLAS database (https://www.proteinatlas.org). We then focused on *Jund* in this study. Flow cytometry analysis confirmed JunD protein expression in hG2-5 cells, regardless of dox treatment (Fig. S8, *E* and *F*). We next examined reported JunD ChIP-seq data using the ChIP-Atlas database (https://chip-atlas.org). JunD binding was observed at the *Csf1* −36.5 kb, *Cxcl10* −20.5 kb, and *Vcam1* −60 kb genomic regions identified in this study (Figs. 7*E*; S8, *G* and *I*). To assess the impact of AP-1 on *Csf1*, *Cxcl10*, and *Vcam1* gene expression upon GATA2 induction, we performed RT-qPCR using the AP-1-specific inhibitor T-5224 (50). Consistent with the RNA-seq results, dox-inducible GATA2 expression significantly upregulated these genes upon TNF-α stimulation (Figs. 7*F*; S8, *H* and *J*). T-5224 treatment did not markedly alter *Gapdh* expression (Fig. S8*K*). Notably, T-5224 treatment reduced *Csf1* and *Cxcl10* expression levels in a dose-dependent manner, and all three genes exhibited statistically significant reduction at 40 μM (Figs. 7*F*; S8, *H* and *J*).

## Discussion

While accumulating evidence suggests a key role for GATA2 in kidney inflammation, the underlying molecular mechanisms remain unclear. In this study, we generated dox-inducible GATA2-expressing cell lines using mIMCD-3 cells. We performed RNA-seq, ATAC-seq, and GATA2 CUT&Tag using this cell line, and ATAC-seq using GATA2-expressing cells from mouse kidney tissues. These experiments demonstrate for the first time that GATA2 directly activates the transcription of kidney inflammation-associated genes, including *Csf1*, *Cxcl10*, and *Vcam1*. Motif analysis revealed that AP-1, NF-κB, and IRF-binding sequences are enriched in the vicinities of the genomic regions where GATA2 increases chromatin accessibility.

Based on these results, we proposed a model to explain the molecular mechanisms by which GATA2 accelerates kidney inflammation (Fig. 7*D*). In this model, GATA2 binds to the CREs of inflammation-related genes and opens the chromatin structure containing the binding sequences of inflammation-induced TFs. This chromatin environment promotes efficient gene transcription *via* inflammation-induced TFs, thereby contributing to kidney inflammation.

Our motif analysis revealed enrichment of AP-1 binding sequences adjacent to GATA2-binding genomic regions in both cell lines and mouse kidney tissues. JunD bound to the *Csf1*, *Cxcl10*, and *Vcam1* loci, and an AP-1 inhibitor attenuated their GATA2-induced upregulation. AP-1, a TF composed of JUN-FOS heterodimers, plays a crucial role in inflammatory responses (51). In vascular endothelial cells, 69% of GATA2-binding peaks overlapped with those of c-JUN (52). In CD4^+^ T cells, primed enhancers showed strong enrichment for both GATA3 and AP-1 motifs (53). Furthermore, GATA2-mediated increased chromatin accessibility facilitated the binding of other TFs, including androgen receptor (AR) and SRY-Box Transcription Factor 9 (Sox9), to CREs in prostate cancer cell lines (54, 55, 56). These studies support our findings and proposed model that GATA2 and AP-1 cooperatively promote kidney inflammation. Further analyses—such as enhanced AP-1 recruitment to the target locus during GATA2 induction and its suppression by T-5224—would contribute to a more detailed molecular understanding of the proposed model.

The *Csf1* gene, encoding macrophage colony-stimulating factor (M-CSF), and the *Cxcl10* gene are both expressed in renal epithelial cells and play key roles in macrophage production and migration (42, 43). We previously found that renal tubular cell-specific *Gata2* deletion mice exhibited a reduced number of F4/80-positive macrophages in the kidney during renal ischemia–reperfusion injury (5). We surmise that decreased levels of *Cxcl10* and *Csf1* partially account for the phenotype observed in renal tubular cell-specific *Gata2* deletion mice. *Vcam1* has been reported to be upregulated in injured renal cells through activation of NF-κB, TNF, and AP-1 pathways (45). *Vcam1* upregulation promotes kidney inflammation by mobilizing immune cells that express its receptor CD49d (46). In addition to the kidneys, GATA2 upregulates *Vcam1* expression in vascular endothelial cells (21, 57). A detailed analysis of GATA2 regulation and its target genes presumably provides further insights into the molecular basis of kidney inflammation.

GATA2 expression promotes inflammation in the whole body and various tissues, including mast cells, vascular endothelial cells, lung cancer, and kidneys (5, 40, 41, 52, 58, 59). In contrast, GATA2 functions as an anti-inflammatory effector in hematopoietic progenitor cells, leukemia cells, and prostate cancer (48, 49, 60, 61, 62). The molecular mechanisms underlying these distinct roles of GATA2 in different cell types remain unanswered questions. TFs regulate chromatin accessibility by interacting with their cofactors. Although it was previously thought that most cofactors functioned ubiquitously, recent findings indicate that they act in a more specific manner, depending on transcriptional timing and cell type (63, 64, 65). Further analysis of GATA2-interacting cofactors offers an intriguing approach to understanding the cell type-specific roles of GATA2 in inflammatory responses.

Here, we showed that chromatin accessibility at *Csf1*, *Cxcl10*, and *Vcam1* was significantly higher in the GATA2-enriched fraction under steady state conditions. These data suggest that GATA2-expressing kidney tissues—specifically principal cells (PCs) in the renal collecting duct—possess a primed chromatin landscape that facilitates transcriptional activation at inflammation-associated gene loci. Although hematopoietic immune cells are considered to be the main drivers of kidney inflammation (3, 4), our results imply that non-hematopoietic PCs also contribute to the inflammatory response. Several studies have implicated the renal collecting ducts as a key source of inflammation in murine models of unilateral ureteral obstruction (66, 67). Renal collecting duct cells have plasticity, and abnormal cell populations derived from the renal collecting duct are increased in patients with CKD (68). Together with our findings, these studies highlight the need for comprehensive analysis of collecting duct cells, which may play previously unrecognized roles in inflammation.

To summarize again, this study represents the first genome-wide analysis of GATA2 occupancy and chromatin accessibility in kidney cells. We have demonstrated that GATA2 induction directly upregulates kidney inflammation-associated gene expression. To date, research on kidney inflammation has primarily focused on hematopoietic immune cells and inflammation-induced TFs as crucial regulators (3, 4, 69). Ideally, our findings will contribute to a deeper understanding of kidney inflammation and support the development of new therapeutic strategies for kidney disease.

## Experimental procedures

### Mice

*Gata2* GFP knock-in mice have been previously described (15, 16) and were maintained in a C57BL/6J genetic background. Wild-type littermates served as controls. Male and female mice (8–12 weeks old) were used in this study. All mice were handled in accordance with institutional regulations and ARRIVE guidelines (Animal Research: Reporting of In Vivo Experiments). All animal experiments were approved by the Animal Experiments Committee of Tohoku Medical and Pharmaceutical University (registration number: 24,006-cn). The primer pairs used for genotyping are listed in Table S1.

### Plasmid construction

The PiggyBac tTS-rtTA dox-inducible gene of interest (GOI) expression vector was designed as shown in Figure S2*A*. The vector was synthesized by VectorBuilder. Human and mouse GATA2 proteins share 97.9% overall homology (470/480 amino acids), with 100% identity in the N-terminal transactivation domain (TAD) and both zinc finger (ZF) domains, and 98.5% in the C-terminal TAD. Thus, their functions are considered nearly equivalent. In contrast, their cDNA sequences show minor differences (1163/1302 nucleotides). To distinguish endogenous from exogenous GATA2, we used human GATA2 cDNA in this study. Human full-length GATA2 cDNA and GATA2 cDNA lacking the zinc finger domain (ΔZF) were synthesized and cloned into a pUC57-Simple vector with additional SalI and NotI restriction sites by GenScript. The synthesized cDNA was digested with SalI and NotI, and then ligated into the PiggyBac tTS-rtTA dox-inducible GOI expression vector. A parental vector without a GOI served as the control construct. The pRP-CAG-hyPBase vector (VB900088–2874gzt) was purchased from VectorBuilder. Additional information about the plasmid is provided in Figure S2*A*.

### Generation of stable cell lines and cell culture

The mIMCD-3, an inner medullary collecting duct (IMCD) cell line, has been previously described (28). In the present study, we used the PiggyBac transposon system to generate a stably transfected cell line (70). mIMCD-3 cells were co-transfected with the transposase pRP-CAG-hyPBase vector and either full-length GATA2 cDNA, ΔZF cDNA, or a control vector using jetPEI (Polyplus). Following puromycin selection, GFP-positive cells were sorted into 96-well plates using FACSAria Fusion (BD Biosciences) to isolate single-cell clones. The strategy for generating stable cell lines is shown in Figure S2*C*. Once established, the stable cell lines were maintained in Dulbecco's Modified Eagle Medium (DMEM)/F-12 (Nacalai Tesque) supplemented with 7% fetal bovine serum (Takara Bio, Shiga, Japan) and 3.0 μg/ml puromycin (Nacalai Tesque). The stable cell lines were treated with 1.0 μg/ml doxycycline (FUJIFILM Wako) to induce GOI and stimulated with 20 ng/ml TNF-α (Thermo Fisher Scientific) to activate inflammatory responses. T-5224 (FUJIFILM Wako), a selective AP-1 inhibitor, was incubated at the indicated concentrations for 1 h prior to analysis.

### Preparation of single-cell suspensions

For cell lines, cells were incubated with Accutase (Nacalai Tesque) for 10 min at 37 °C, then dissociated by gently pipetting to prepare single-cell suspensions. For mouse kidney tissues, kidneys were minced with scissors and incubated in Hanks' balanced salt solution (HBSS; FUJIFILM Wako) containing 15 mM HEPES (Nacalai Tesque), 0.5% BSA (Sigma-Aldrich), 1.0 mg/ml Collagenase I (Worthington Biochemical Corporation), 1.0 mg/ml Dispase II (FUJIFILM Wako), and 0.2 mg/ml DNase I (Sigma-Aldrich) for 1 h at 37 °C in a shaking incubator. Following incubation, the kidney tissues were gently pipetted, drawn into a syringe with a 21–25G needle, and filtered through a 40 μm filter. The resulting single-cell suspensions were used for subsequent experiments.

### Flow cytometry analysis and cell sorting

Single-cell suspensions from cell lines were resuspended in PBS containing 2% FBS and 2 mM EDTA (FACS buffer). Single-cell suspensions of mouse kidneys were resuspended in HBSS containing 15 mM HEPES, 0.5% BSA, and 2 mM EDTA. Red blood cells (RBCs) were lysed in RBC lysis buffer (Thermo Fisher Scientific). Intracellular staining of GATA2 and JunD was performed using the eBioscience Foxp3/Transcription Factor Staining Buffer Set (Thermo Fisher Scientific), according to the manufacturer's instructions. Flow cytometry and cell sorting were conducted using a BD LSRFortessa X-20 (BD Biosciences) or FACSAria Fusion (BD Biosciences) as previously described (71, 72). Dead cells were excluded from analyses using a Fixable Viability Dye (Thermo Fisher Scientific). Data analysis was performed using FlowJo software (BD Biosciences). The antibodies and lectin used in the flow cytometry analysis are listed in Table S2, and the gating strategies are shown in Figure S9.

### Western blotting

Western blotting of whole-cell extracts was performed as previously described (73). Proteins were detected using an ECL Prime Kit (GE Healthcare) on a Amasham Imager (GE Healthcare). The antibodies used for western blotting are listed in Table S2.

### Fluorescence imaging

GFP and tdTomato fluorescent images were acquired using a BZ-9000 fluorescence microscope (Keyence).

### Quantitative real-time PCR (qPCR)

Total RNA was extracted using an ISOGEN II (FUJIFILM Wako) or Agecourt RNAadvance Tissue Kit (BECKMAN COULTER) according to the manufacturer’s instructions. cDNA was synthesized using ReverTra Ace qPCR RT Master Mix with gDNA Remover (TOYOBO). Quantitative real-time PCR was performed using THUNDERBIRD Next SYBR qPCR Mix (TOYOBO) on a CFX96 Touch Detection System (Bio-Rad Laboratories). The primers used for the qPCR are listed in Table S1.

### DNase I chromatin accessibility analysis

DNase I Chromatin Accessibility Analysis was performed as previously described with minor modifications (56, 74). qPCR was performed as described above. Chromatin accessibility was calculated using the following formula: 2^/ (CT treated with DNase I–CT untreated). Higher values indicate that the DNA is more susceptible to degradation by DNase I, representing increased chromatin accessibility. The primers used in this assay are listed in Table S1.

### RNA-sequencing

Four groups of hG2-5 cells were subjected to RNA sequencing (RNA-seq) analysis in triplicate (Fig. 3*A*). Cells were gently fixed using CellCover (Anacyte Laboratories), and only viable cells were sorted using a FACSAria Fusion (BD Biosciences). Total RNA was extracted from sorted cells using ISOGEN II. RNA concentration was measured using the Qubit RNA High Sensitivity Assay on a Qubit 4 Fluorometer (Thermo Fisher Scientific). RNA integrity was confirmed using High Sensitivity RNA ScreenTape (Agilent Technologies) on an Agilent 4200 TapeStation System (Agilent Technologies). The library was prepared using a NEBNext Poly(A) mRNA Magnetic Isolation Module and NEBNext Ultra II Directional RNA Library Prep Kit (New England Biolabs), and sequencing was performed on an Illumina NovaSeq 6000 by Rhelixa, generating 150-base pair paired-end reads. Once the FASTQ files were obtained, trimming was performed using Fastp (75), and transcripts per million (TPM) were quantified using Salmon (76) with the mm10 genome. These processes were automated using the RaNA-Seq bioinformatics tool (77). Clustering, gene set enrichment analysis (GSEA), and heatmap visualization were performed using the RNAseqChef bioinformatics tool (78). The TPM values for mIMCD-3, NIH3T3, and P815 were obtained using the GEO RNA-seq Experiments Interactive Navigator (79) (GREIN; https://www.ilincs.org/apps/grein/?gse=).

### Assay for Transposase-accessible chromatin using sequencing (ATAC-seq)

ATAC-seq has been described previously (34, 35). Cell lines and mouse kidney tissues were lightly fixed in 0.1% formaldehyde for 2 min at room temperature. A total of 50,000 to 100,000 viable cells were sorted using a FACSAria Fusion (BD Biosciences). Isolation of intact nuclei, tagmentation, and PCR amplification were performed using an ATAC-seq kit (Active Motif) according to the manufacturer's instructions. Tagmented DNA was incubated overnight at 65 °C in 50 mM Tris-HCl buffer containing 1% SDS, 1 mM EDTA, 200 mM NaCl, and 0.1 mg/ml proteinase K for reverse crosslinking. Purified DNA was obtained using a NucleoSpin Gel and PCR Clean-up Kit (Takara Bio) and subsequently amplified by PCR. PCR-amplified DNA was sequenced using an Illumina NovaSeq X Plus by Novogene. After obtaining the FASTQ files, the following analyses were automatically performed using the Basepair NGS Data Analysis Platform (Basepair): trimming with Fastp (75), alignment to the mm10 genome using Bowtie2 (80), peak calling with MACS2 (81, 82), and motif analysis using HOMER (83). Experiments were performed in duplicate for each sample.

### Cleavage Under Targets & tagmentation (CUT&Tag)

The CUT&Tag assay has been previously described (36). Cell lines were lightly fixed in PBS with 0.1% formaldehyde for 2 min at room temperature. A total of 100,000 to 125,000 viable cells were sorted using FACSAria Fusion (BD Biosciences). CUT&Tag was conducted using the CUTANA CUT&Tag Kit (EpiCypher) according to the manufacturer's instructions. Libraries generated from the CUT&Tag experiment were sequenced on an Illumina NovaSeq X Plus by Novogene. After obtaining the FASTQ files, analyses were performed automatically using the Basepair NGS Data Analysis Platform as described for ATAC-seq. Experiments were performed in duplicate or triplicate for each sample. The antibodies used in the CUT&Tag analysis are listed in the Table S2.

### Quantification and statistical analyses

Comparisons between the two groups were conducted using unpaired t-tests. Comparisons among multiple groups were conducted using one-way analysis of variance (ANOVA) followed by Tukey’s *post hoc* test. Multiplicity was adjusted using the Benjamini–Hochberg method. The statistical methods used in each experiment are described in detail in the figure legends. Unless otherwise noted, statistical significance was defined as *p* < 0.05. Data are presented as means ± SD, as described in the figure legends. Data management and statistical analysis were performed using Microsoft Excel (Microsoft Corp.) and GraphPad Prism 8 (GraphPad Software).

## Data availability

All data generated or analyzed in this study are included in this published article and its Supporting Information file. RNA-seq, ATAC-seq, and CUT&Tag data generated in this study have been deposited with links to BioProject accession number PRJNA1255840 in the NCBI BioProject database (https://www.ncbi.nlm.nih.gov/bioproject/). Additional data are available upon request from Jun Takai (j-takai@tohoku-mpu.ac.jp).

## Supporting information

This article includes supporting information.

## Conflict of interest

The authors declare that they have no conflicts of interest with the contents of this article.
