# Supplementary material for: Induction of the zinc finger transcription factor GATA2 promotes kidney inflammation-related gene expression
Source: J Biol Chem. 2025 Jun 16;301(7):110372. doi: 10.1016/j.jbc.2025.110372 (PMC12281529; doi:10.1016/j.jbc.2025.110372)
Supplement: Supporting information [file mmc1.pdf]

Supporting Information

Title
Induction of the zinc finger transcription factor GATA2 promotes kidney inflammation-related gene expression

Authors
Jun Takai[1]*, Hinata Ueki[1#] and Satoshi Uemura[1#].
[1]Division of Medical Biochemistry, Tohoku Medical and Pharmaceutical University, School of Medicine, Sendai.
* For correspondence: Jun Takai, j-takai@tohoku-mpu.ac.jp
[#] These two authors contributed equally to this work

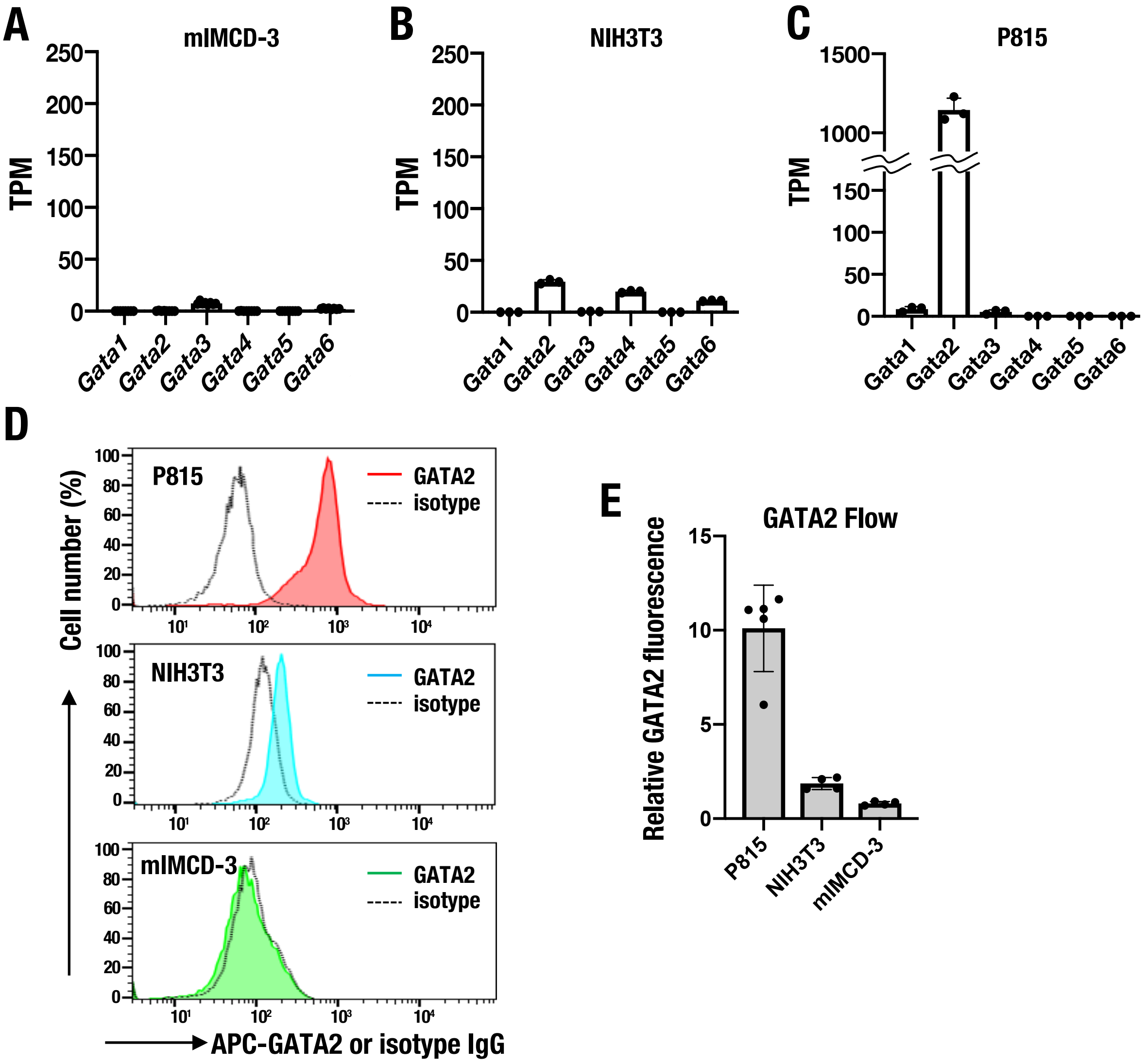

Figure S1. GATA2 expression levels in the mIMCD-3 cell line. (A–C) mRNA expression levels of all GATA transcription factors in mIMCD-3 (n = 4), NIH3T3 (n = 3), and P815 (n = 3) cells. Transcripts per million (TPM) values for each cell line were obtained using the GEO RNA-seq Experiments Interactive Navigator (GREIN; https://www.ilincs.org/apps/grein/?gse=). Gene Expression Omnibus (GEO) accession IDs are as follows: mIMCD-3, GSE171573; NIH3T3, GSE129593; and P815, GSE181773. (D) Representative histograms of GATA2 fluorescence and isotype IgG fluorescence analyzed by flow cytometry. (E) Relative GATA2 fluorescence levels quantified by flow cytometry (n = 5 for P815; n = 4 for NIH3T3 and mIMCD-3). Values were normalized to the isotype IgG control. Data from independent replicates are plotted in the bar graph. All data are presented as means ± SD in the graph.

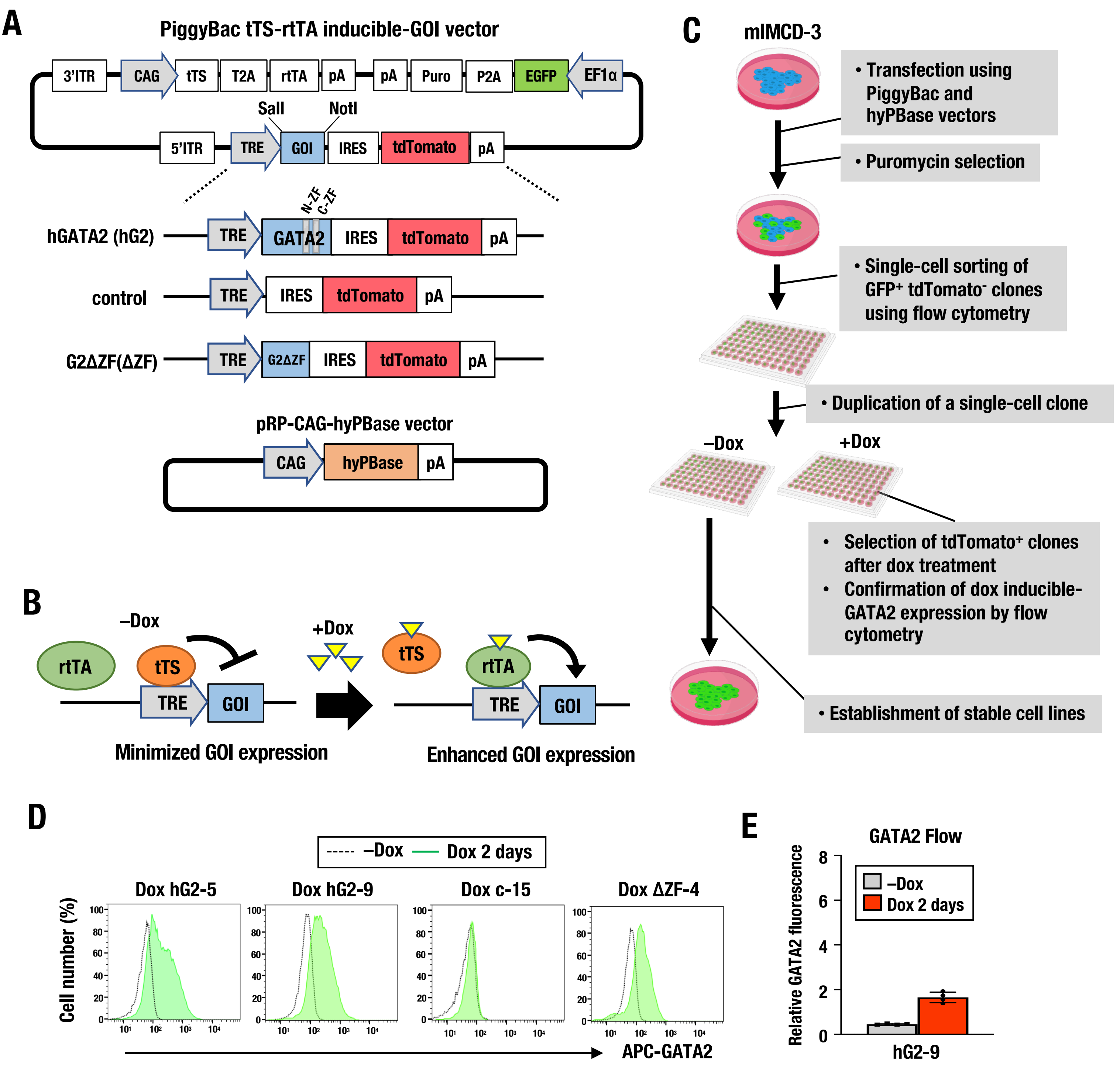

Figure S2. Schematic diagrams of a stable cell line with a doxycycline (dox)-inducible GATA2 expression vector. (A) Constructs of the PiggyBac tTS-rtTA inducible-GOI vector and the pRP-CAG-hyPBase vector used in this study. SalI and NotI restriction sites are located at both ends of the GOI for cDNA insertion. The hGATA2, control, and G2ΔZF constructs used to generate stable cell lines are shown. Abbreviations: ITR, inverted terminal repeats; tTS, tetracycline transcriptional silencer; rtTA, reverse tetracycline-controlled transactivator; TRE, Tet response element promoter; GOI, gene of interest; N-ZF, N-terminal zinc finger; C-ZF, C-terminal zinc finger; hyPBase, hyperactive PiggyBac transposase. (B) Overview of the tTS-rtTA combination system. In the absence of dox, tTS represses GOI expression. Upon dox treatment, tTS is inactivated, and rtTA activates GOI expression. This system thus minimizes leakage in the absence of dox and enhances GOI induction efficiency. (C) Flowchart outlining the procedure for generating a stable cell line. Transposase (hyPBase) recognizes the two ITR sequences in the PiggyBac vector and cleaves the DNA between them. The excised sequence is then integrated into the host genome. (D) Representative histograms of GATA2 fluorescence in each construct, analyzed by flow cytometry without dox (–Dox) or 2 days after dox treatment. (E) Relative GATA2 fluorescence levels in hG2-9 cells, quantified by flow cytometry (n = 4). Values were normalized to the isotype IgG control. Data from independent replicates are plotted in the bar graph. All data are presented as means ± SD in the graph.

Takai et al, Figure S2

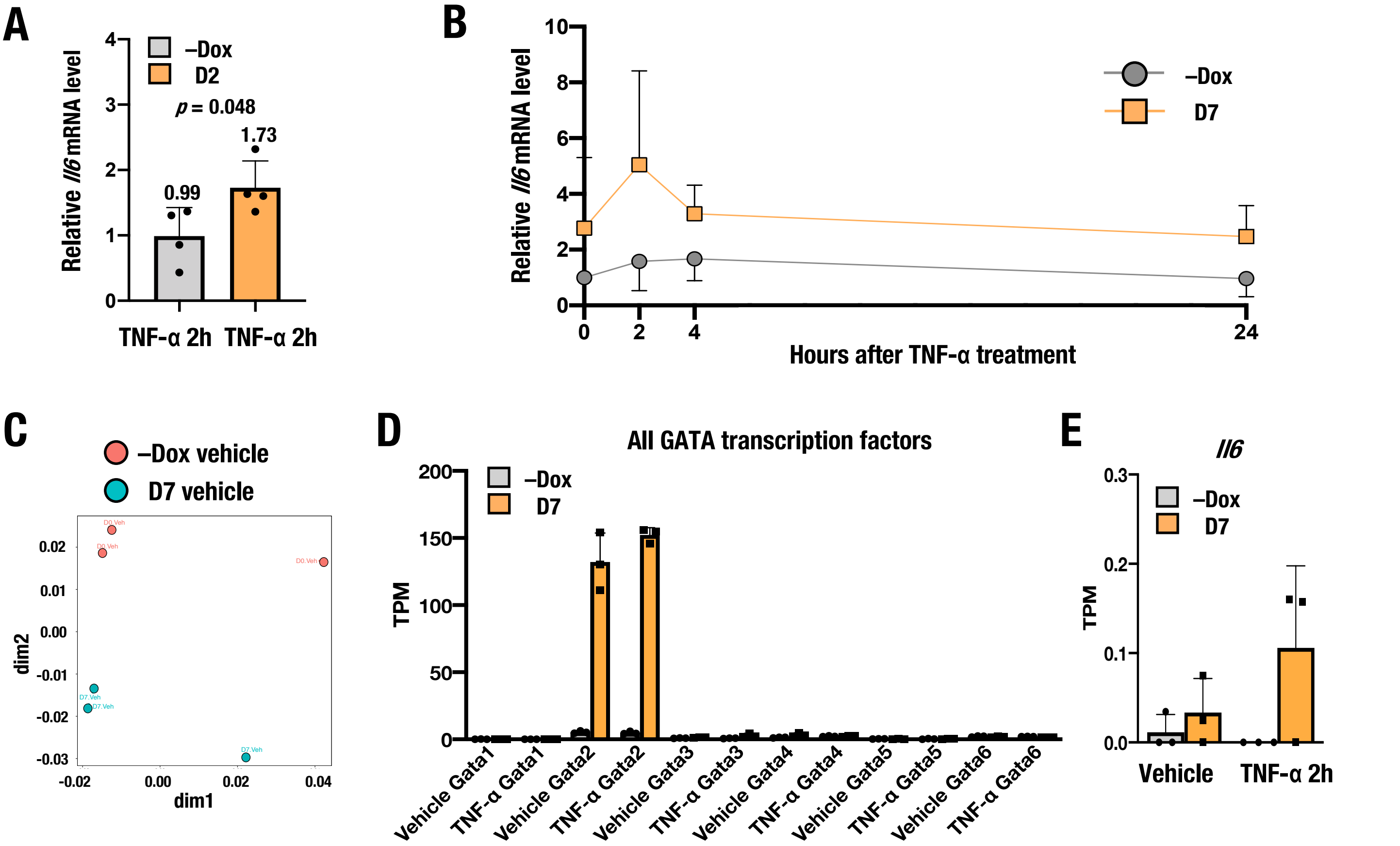

F

D7 vehicle vs –Dox vehicle

Gene_set_name	ES	NES	*q*-value
Epithelial_ mesenchymal_ transition	0.47	1.51	0.0136
Myc_targets_v1	-0.53	-1.84	0.0000131

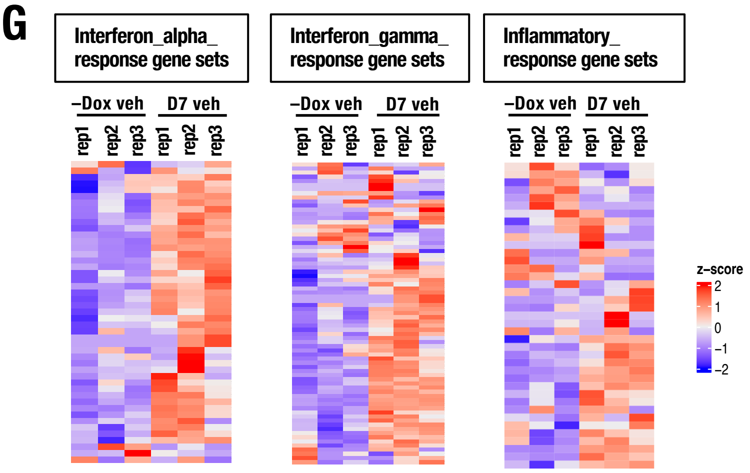

Figure S3. Additional data from manual RT-qPCR and RNA sequencing.
(A) Relative *Il6* mRNA levels in hG2-5 cells after TNF-α stimulation without dox (–Dox) and 2 days after dox treatment (D2), quantified by RT-qPCR (n = 4 per group). *p*-values were calculated using unpaired t-tests. (B) Time-course analysis of *Il6* mRNA levels in hG2-5 cells after TNF-α stimulation under –dox and +dox 7 days (D7), quantified by RT-qPCR. (C) Clustering of vehicle-treated groups using multidimensional scaling. (D) mRNA expression levels of all GATA transcription factors, quantified by RNA-seq (n = 3 per group). (E) mRNA expression levels of *Il6*, quantified by RNA-seq (n = 3 per group). (F) GSEA comparing D7 vehicle and –Dox vehicle. Gene sets with *q*-values (multiple testing adjusted *p*-values) below 0.05 are listed. Abbreviations: ES, enrichment score; NES, normalized enrichment score. (G) Heatmaps displaying gene sets for Interferon_alpha_response, Interferon_gamma_response, and Inflammatory_ response in D7 vehicle and –Dox vehicle. Data from independent replicates are plotted in the bar graph. All data are presented as means ± SD in the graph.

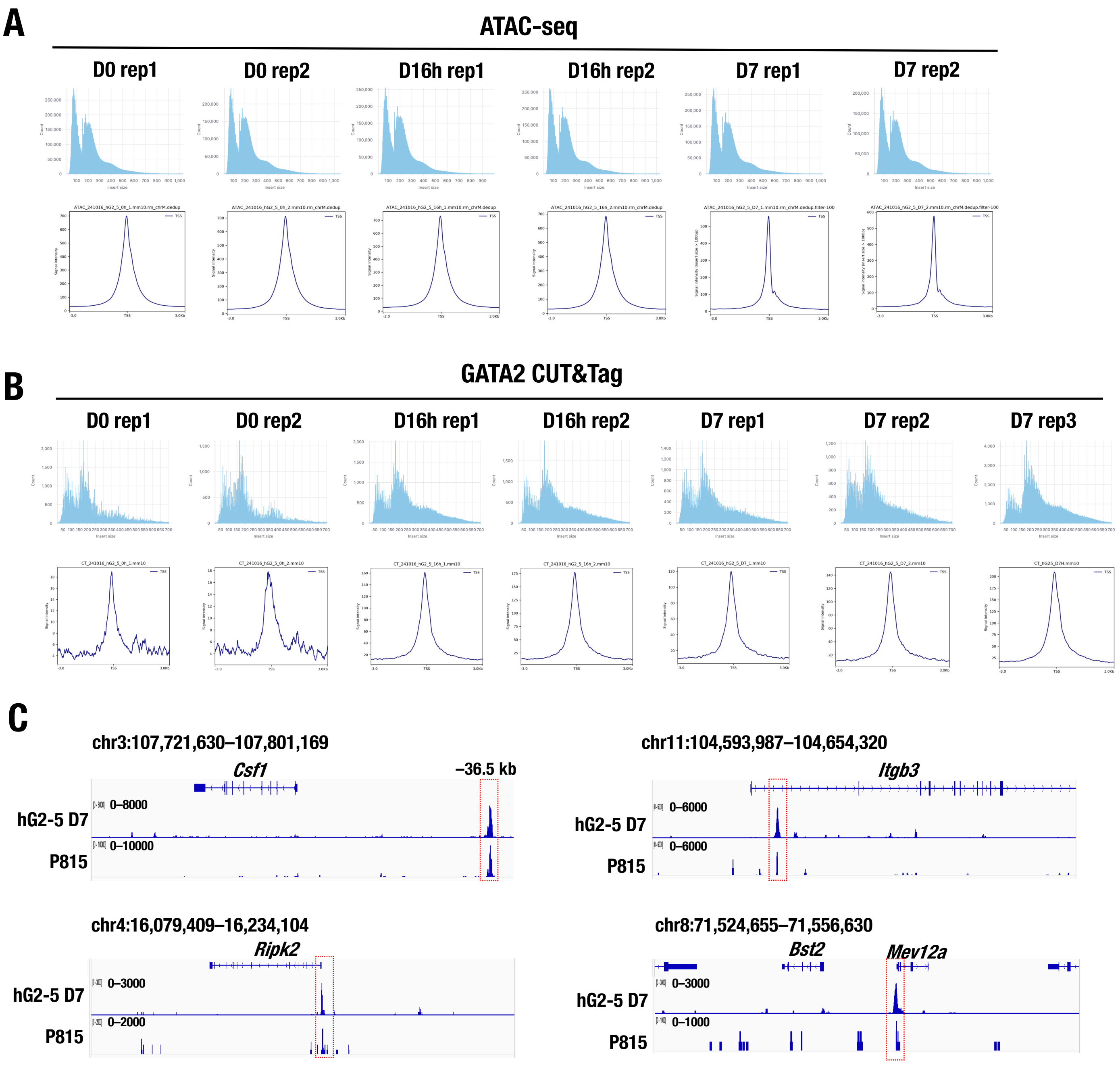

Figure S4. QC results of GATA2 CUT&Tag and ATAC-seq in hG2-5 cells. (A, B) TSS signal intensity and insert size distribution from ATAC-seq data (A, upper panel) and GATA2 CUT&Tag (B, lower panel) in the hG2-5 cell line. Note that sharp peaks around the TSS and nucleosome ladders were observed in all samples except D0 rep1 and D0 rep2 of GATA2 CUT&Tag. (C) Representative GATA2 binding peaks shared between hG2-5 D7 and P815 cells.

A

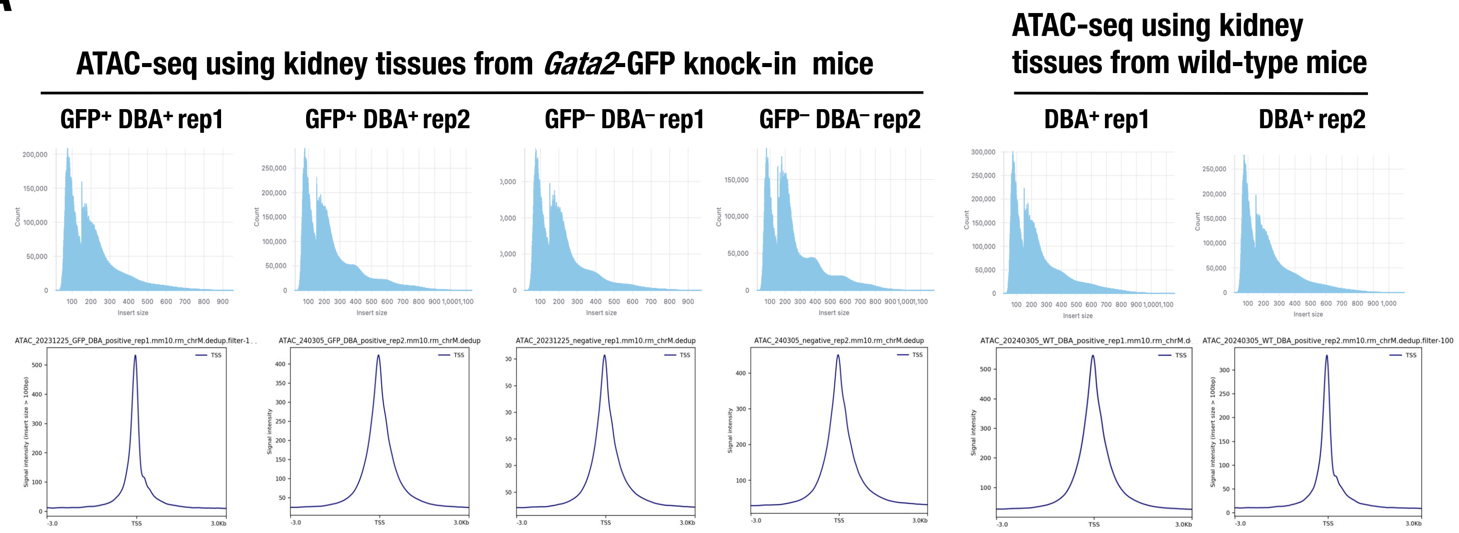

Figure S5. QC results of ATAC-seq using mouse kidney tissues. (A) TSS signal intensity and insert size distribution from the ATAC-seq data of mouse kidney tissues. Note that sharp peaks around the TSS and nucleosome ladders were observed in all samples.

Takai et al, Figure S5

A

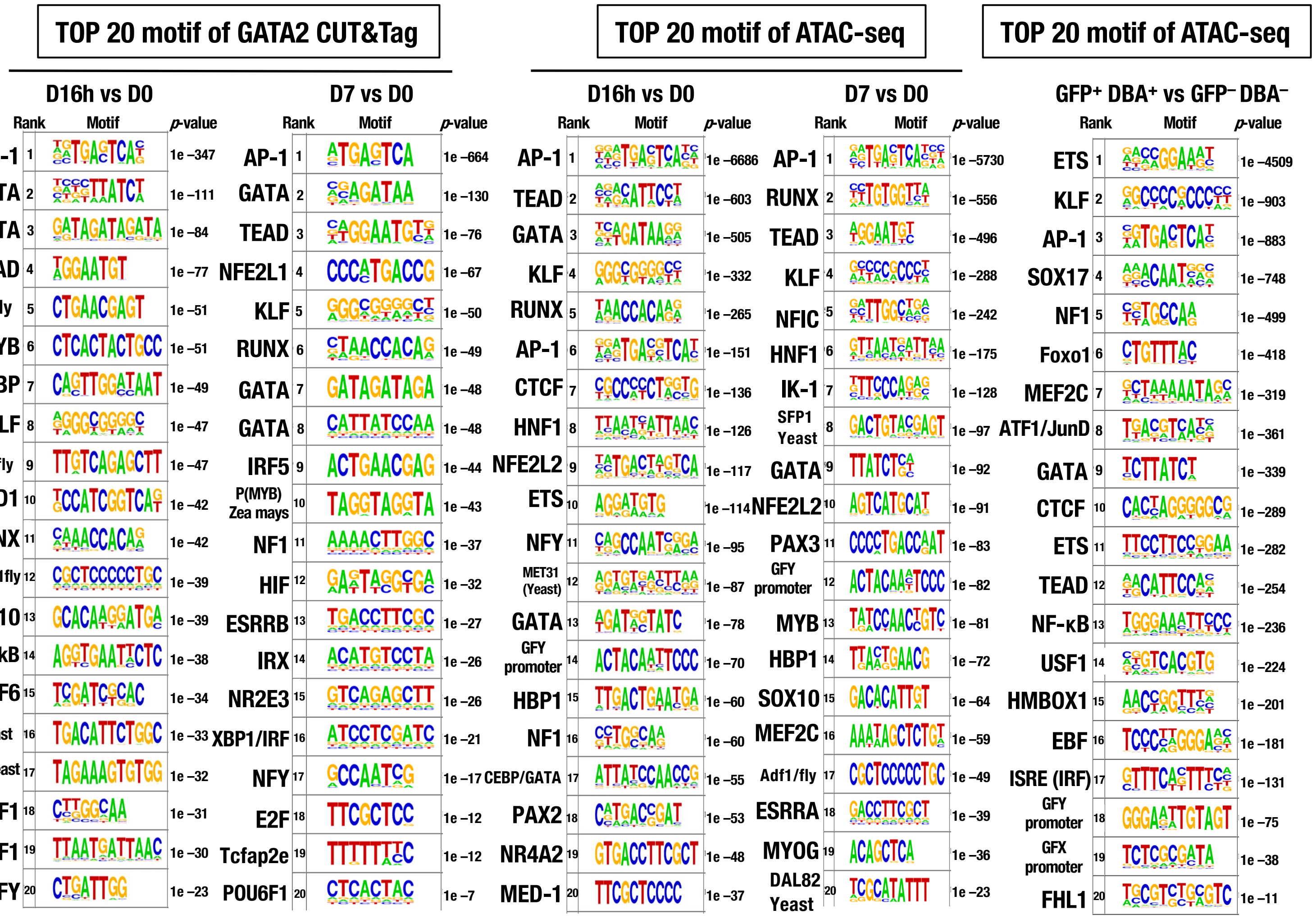

Figure S6. Top 20 transcription factor binding motifs specific to GATA2-expressing cells. (A) The top 20 transcription factor binding motifs specific to GATA2-expressing cells, identified from all CUT&Tag and ATAC-seq data generated in this study. Motifs were ranked in descending order of *p*-values using HOMER. *p*-values were calculated using a hypergeometric test.

Takai et al, Figure S6

A *Cxcl10* –20.5 kb

→ AP-1 → GATA
ATAGAAGACGGATGACTCATCCTATGGAAAAGATTGCCAGAAGTCCAGAATAACGTGCCGGGACTGAAGAGAGCGATAGCCAAATGCAGATCCTGTAACCTTATGGCAGTACAACACCTG

NF-κB ←
CTGTTCTTTTCTATAACAACACAGTCATCTTAGAGAGCGTAGTGAATTTTGTTATTGTTAAGACATTACCACTGTGATTTCCTGACATTACAATACTGACCCAGTAGTCAGGAAAACCCTATA

→ IRF
CACTGTGTTCAGAGTGTCTGTCAGACCCTGAGTAAGACCACAATGAAGGAAACAAATAGAAACTAGAAAGAAATCATAAAAACACAAAAGTCCACGAAAACACAGAAAAAAAAATGTGCAT

TTTTTTTGTTGCCTAAAAGAACCCCAAAATGAGGGTGATTTTTGAAGAAGAAGAAAAAAAAAAGTTGTTTCTGAACTTAAGCTTAATCTTCCTATAATTTGACATAGAAAATGTTTTCAAAACC

GATA ←
CGCATTCCGCTTATGCTGTCTGGTATCTGAAATAGATCTGTCAGGGGGTCACATTTTATAAGCACCACTTCGTGTTTGATGTGTGCTGTCCCAGACATTACAGCATTGGGCATGCGCACAGGA

→ AP-1
AGGAGGGGCCGTTGCAGTAGTCCCCACTGTCTGACTGACCACCCCATGCTTGAGAGAACCATTATGAGTCATGGTTC

B *Vcam1* –60 kb

→ AP-1 → GATA
TATGCCTCACAAATAGAAGGCCTGAGCAATGTGTTTAAAAAGCAAATATAAAGAGCAGCCAGTCAATATTATTTGATATGTCTGTCTTAATAAAATCATTTGATGGGCCACATCAGTGGAGGC

→ NF-κB → AP-1
AAAATGATCCAGGATGAACTACAGTAGGGCTTATGAACTTGTGAGTGGGCATTCACTTTAACTTGATCCCATGGTGAAAGCAATCCTACCCATTGACGCTGGCACCTTCAACGCCTTAATCAT

AP-1 ← → AP-1 IRF ←
TTCTGGTTAGTCATGTGGATTTTCATATGACTAACTGCCCTTGGCAGGCAACTTAAGCAAGTTTATTTCACAGTCAGCAGCCAAGGGCTTCCTATTTTCAGTTTTACCCTCAATGGGAACCAGC

CATCCAGCTGAGCATTCAAGACCTGTTCTTTAGAGTTCACTAGGACCAAGAGAACTCAGTCCACAGGCCAGTGGACTGTGTACAAGTCTTCTCAAAACAACCACCTTTAATAACCTGGACCA

→ GATA
GAGTTTATAGAGAACAAATTTCCCTCTTTAGCTCTGATACATATCTCTAGGGTGATTTGAGTGTCAAACGCCCTCTTCCTGGGTCCATAAATCATAAACTCAGGATTTACTTAAGTAGCTTAGT
GATA ←
CATATGCTAAATATTACAGACAGGAAAGATCTGAGCACCAGAGGATGGGAAGGCCGGGGTGAACTCTTGCCCGGCCTTTCCCCTCATCTAGACTGAGCAACAGAACAAAGTAGTCTACCTGG

GATA ←
TTACATGATCGGCAACTCTGAAATTCAGAGACTCTATACAAGTGTACAGATTCCCAAAGTTGTTTTTCTCTGTAAAACACATATCCTGCTCTCACTTGGACAACTTTTACGATGACAGACT

Figure S7. Nucleotide sequences at the *Cxcl10* –20.5 kb and *Vcam*1 –60 kb genomic regions. Nucleotide sequences at the *Cxcl10* –20.5 kb and *Vcam1* –60 kb genomic regions around the GATA2-binding peak identified by CUT&Tag. Binding motifs for inflammation-induced TFs and GATA are indicated.

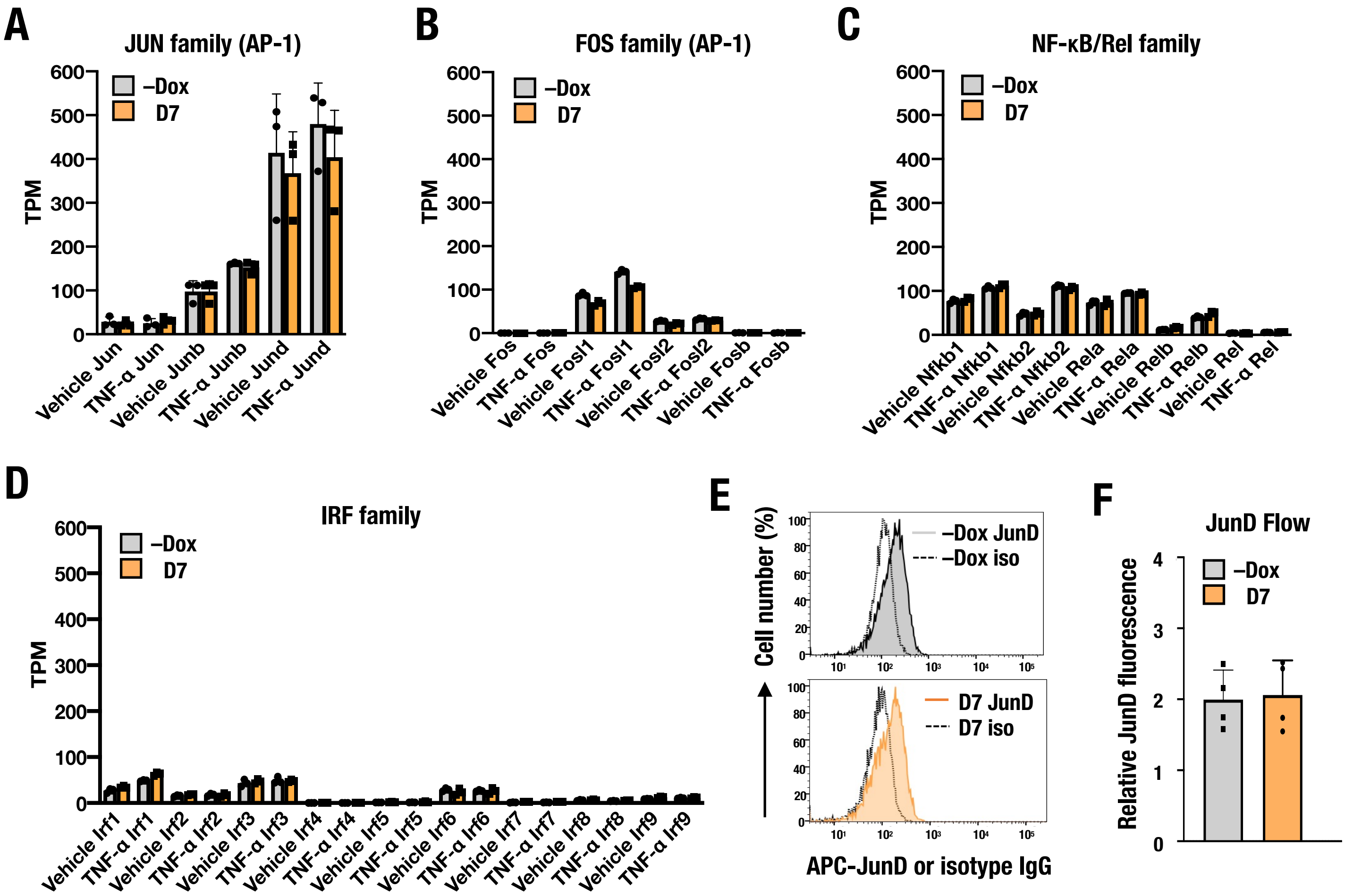

Figure S8. Involvement of AP-1 in the up-regulation of kidney inflammation-associated genes by GATA2 induction. (A–D) mRNA expression levels of the JUN family, FOS family, NF-κB/Rel family, and Interferon regulatory factor (IRF) family in hG2-5 cells, analyzed by RNA-seq (n = 3 per group). (E) Representative histograms of JunD and isotype IgG control (iso) fluorescence in hG2-5 cells without dox (–Dox) and 7 days after dox treatment (D7). (F) Relative JunD fluorescence levels in hG2-5 cells, quantified by flow cytometry (n = 4). Values were normalized to the isotype IgG control. Data from independent replicates are plotted in the bar graph. All data are presented as means ± SD in the graph.

(figure and legend continued on next page)

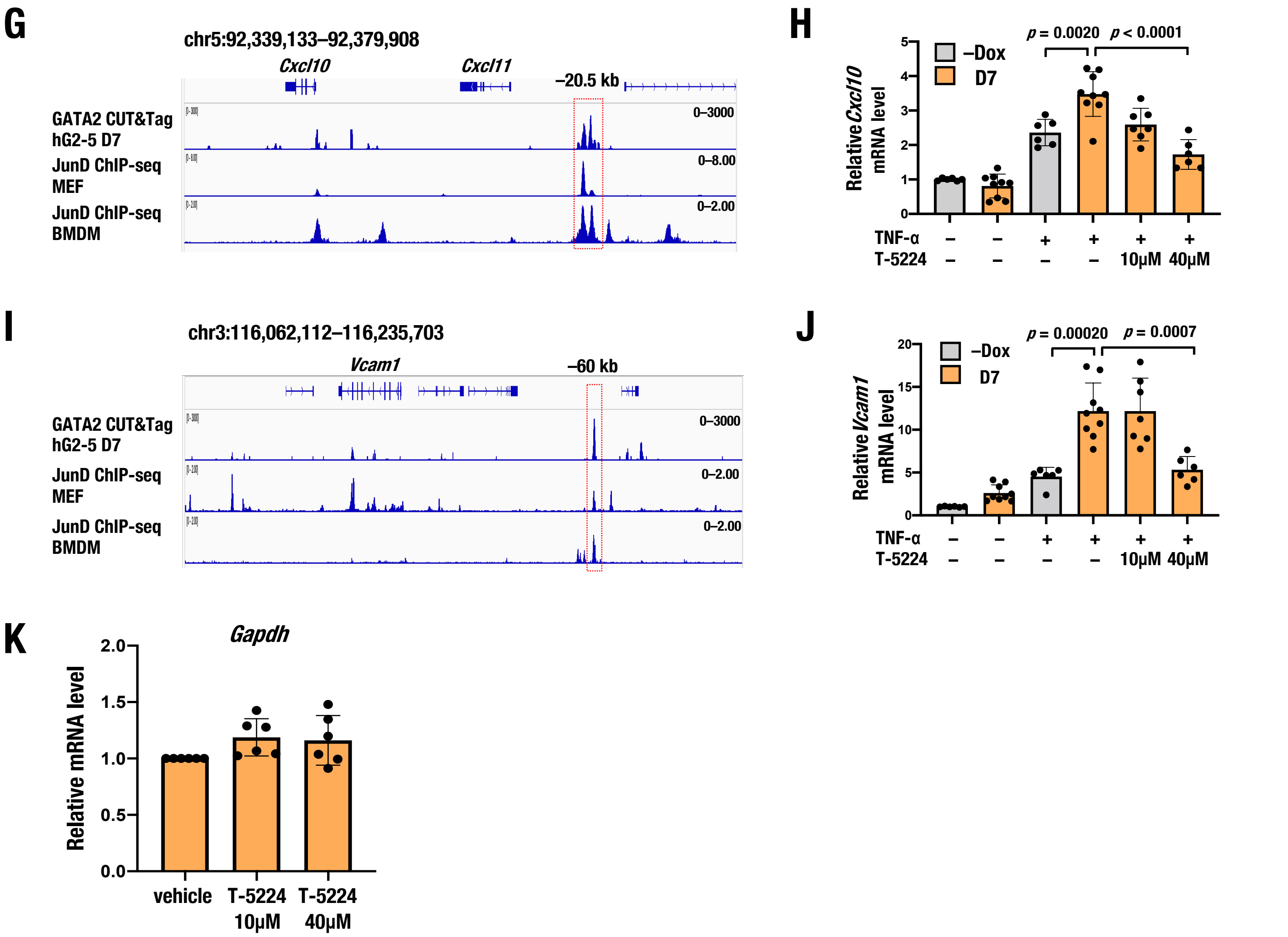

Figure S8. Involvement of AP-1 in the up-regulation of kidney inflammation-associated genes by GATA2 induction. (G, I) Representative GATA2 and JunD binding peaks at the *Cxcl10* and *Vcam1* loci. GATA2 CUT&Tag data from hG2-5 cells at D7 and previously reported JunD ChIP-seq data (SRX13793008 and SRX2901296) are shown. Abbreviations: MEF, mouse embryonic fibroblast; BMDM, bone marrow-derived macrophage. (H, J) Relative *Cxcl10* and *Vcam1* mRNA levels at –Dox and D7 in hG2-5 cells, quantified by RT-qPCR (n = 6 for –Dox group and n = 6–9 for D7 group). Cells were treated with TNF-α (20 ng/ml, 2 h) and the AP-1 inhibitor T-5224 (10 or 40 μM, 1 h) prior to analysis. *p*-values in (H, J) were calculated using one-way ANOVA followed by Tukey's post hoc test. (K) Relative *Gapdh* mRNA levels in hG2-5 cells, quantified by RT-qPCR (n = 6 per group). Cells were treated with vehicle or the AP-1 inhibitor. mRNA levels were normalized to *Polr2a*, with the vehicle-treated group defined as 1.0. Data from independent replicates are plotted in the bar graph. All data are presented as means ± SD in the graph.

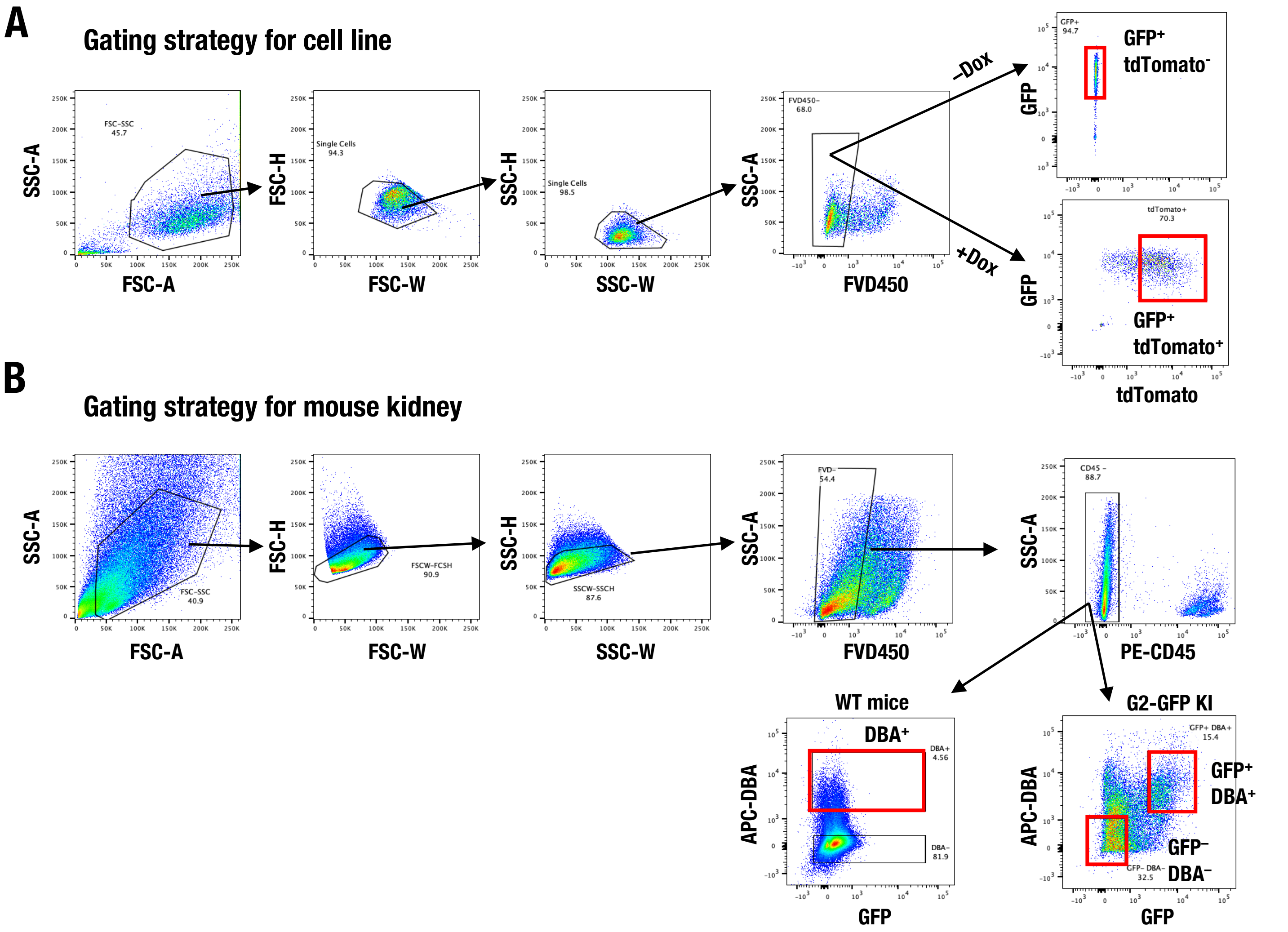

Figure S9. Gating strategy for flow cytometry. (A, B) Gating strategy for cell lines (A, upper panel) and mouse kidney tissues (B, lower panel). The fractions used for the analysis are circled in red.

Table S1. Primer sequences used in this study

Name	Sense primer	Antisense primer	Assay
Gata2-GFP KI	CTGAAGTTCATCTGCACCACC	GAAGTTGTACTCCAGCTTGTGC	Genotyping
hGATA2 (human specific)	TGTCAGACGACAACCACCAC	TCTCCTGCATGCACTTTGAC	RT-qPCR
Il6	TACCACTTCACAAGTCGGAGGC	CTGCAAGTGCATCATCGTTGTTC	RT-qPCR
Csf1	CTTCATGCCAGATTGCCTTT	ATGGAAAGTTCGGACACAGG	RT-qPCR
Cxcl10	CTATCCTGCCCACGTGTTGA	CCATCCACTGGGTAAAGGGG	RT-qPCR
Vcam1	CTGGGAAGCTGGAACGAAGT	GCCAAACACTTGACCGTGAC	RT-qPCR
Polr2a	CTGGACCCTCAAGCCCATACAT	CGTGGCTCATAGGCTGGTGAT	RT-qPCR
Gapdh	GAGATGATGACCCTTTTGGC	GTCGTGGAGTCTACTGGTGTCTT	RT-qPCR
Il6 promoter	AGG GCT AGC CTC AAG GAT GAC	GTG GGG CTG ATT GGA AAC CT	DNase I Chromatin accessibility analysis
Gata2 -27.7 kb	TGCCATGCCGGATATATTTTG	ACTAGCACGTGTGGCACAGTG	DNase I Chromatin accessibility analysis

Table S2. Antibodies used in this study and reagent used in flowcytometry

Name	Source	Clone or catalog#	Assay
GATA2 (mouse monoclonal)	Perseus Proteomics	B9922A	Flow, WB, CUT&Tag
β-actin (mouse monoclonal)	Sigma-Aldrich	AC-15	WB
Peroxidase-conjugated goat anti-mouse IgG (H+L), Secondary Antibody	Thermo Fisher	# 31430	WB
GATA2 (rabbit monoclonal)	Cell Signaling Technology	E9T6F	CUT&Tag
GATA2 (rabbit polyclonal)	Proteintech	11103-1-AP	CUT&Tag
IgG (rabbit polyclonal)	Epicypher	13-0042	CUT&Tag
Anti-Rabbit Secondary Antibody (Goat mixed Monoclonal)	Epicypher	13-0047	CUT&Tag
Anti-Mouse Secondary Antibody (Goat mixed Monoclonal)	Epicypher	13-0048	CUT&Tag
PE-CD45 (rat monoclonal)	eBiocience	30-F11	Flow
Anti-Mouse Secondary Antibody, APC (Goat polyclonal)	eBiocience	17-4010-82	Flow
Biotin-DBA	Vector Laboratories	B-1035-5	Flow
SAV-APC	eBiocience	17-4317-82	Flow
Fixable Viability Dye eFluor™ 450	eBiocience	65-0863-14	Flow
Normal mouse IgG	Santa Cruz	sc-2025	Flow
Rabbit (DA1E) mAb IgG XP® Isotype Control	Cell Signaling Technology	DA1E	Flow
Alexa Fluor® 647 Donkey anti-rabbit IgG (Donkey polyclonal)	Biolegend	Poly6047	Flow
APC anti-mouse CD106 (VCAM1) Antibody	Biolegend	429	Flow
JunD (rabbit monoclonal)	Cell Signaling Technology	D17G2	Flow
